# Exploiting deep neural network and long short-term memory method-ologies in bioacoustic classification of LPC-based features

**DOI:** 10.1371/journal.pone.0259140

**Published:** 2021-12-23

**Authors:** Cihun-Siyong Alex Gong, Chih-Hui Simon Su, Kuo-Wei Chao, Yi-Chu Chao, Chin-Kai Su, Wei-Hang Chiu

**Affiliations:** 1 Department of Electrical Engineering, Chang Gung University, Taoyuan, Taiwan; 2 Department of Ophthalmology, Chang Gung Memorial Hospital, Linkou Branch, Taoyuan City, Taiwan; 3 Department of Mechanical Engineering, National Cheng Kung University, Tainan, Taiwan; 4 Department of Public Health, National Taiwan University, Taipei, Taiwan; 5 Fudan High School, Taoyuan, Taiwan; Vietnam National University, VIET NAM

## Abstract

The research describes the recognition and classification of the acoustic characteristics of amphibians using deep learning of deep neural network (DNN) and long short-term memory (LSTM) for biological applications. First, original data is collected from 32 species of frogs and 3 species of toads commonly found in Taiwan. Secondly, two digital filtering algorithms, linear predictive coding (LPC) and Mel-frequency cepstral coefficient (MFCC), are respectively used to collect amphibian bioacoustic features and construct the datasets. In addition, principal component analysis (PCA) algorithm is applied to achieve dimensional reduction of the training model datasets. Next, the classification of amphibian bioacoustic features is accomplished through the use of DNN and LSTM. The Pytorch platform with a GPU processor (NVIDIA GeForce GTX 1050 Ti) realizes the calculation and recognition of the acoustic feature classification results. Based on above-mentioned two algorithms, the sound feature datasets are classified and effectively summarized in several classification result tables and graphs for presentation. The results of the classification experiment of the different features of bioacoustics are verified and discussed in detail. This research seeks to extract the optimal combination of the best recognition and classification algorithms in all experimental processes.

## 1. Introduction

In nature, communication between animals entails the transmission of specific information between individuals of one or different species to invoke specific behaviors [1]. Therefore, considerable work has focused on the study of animal behavior based on acoustic feature analysis [2, 3]–even those abiotic signals have been studied. Several available adaptive theories analytical methods can be used to extract hidden information conveyed by any sound [4]. For example, the sound of human breathing, the release of vibration energy from objects, or the abnormal automobile driving sound characteristics may implicitly indicate the existence of some abnormal problems [5, 6]. Different acoustic characteristics represent dynamic behavior characteristics under actual conditions. The sound characteristics of each animal reflect the actual state of animal behavior, and thus reveal information about different behaviors [7], and the sound information communicated by a large number of animals can be automatically and systematically measured and monitored in nature.

By collecting and analyzing the characteristics of animal communication sounds of different species, this research provides a more benefit and convenient way to monitor the dynamic behavior of specific animal species, avoiding time-consuming manual monitoring and analysis [8]. The application of bioacoustic monitoring technology is very effective in identifying existing species, especially in the case of species for which limited data is available [9]. Many well-known research cases have established that acoustic signal data can be effectively collected and digitally filtered feature identification [10, 11]. The application of signal comparison and recognition for bioacoustics includes well-trained artificial listening recognition or classification by multi-channel spectrogram observation. Detection based on collected signals depends on sensor signal measurement and acquisition using classifier algorithms such as machine learning. Well-trained professional observers can distinguish subtle spectrogram features, and then can identify relevant sound features in the surrounding environment [12]. The time series classification and calculation method has emerged as a popular artificial intelligence research topic.

Most supervised and unsupervised algorithms are typically applied to dynamic time series signals [13]. Automatic animal sound detection and recognition from audio recordings is gradually becoming an emerging topic in bioacoustics [14]. Technically speaking, bioacoustic features and classification, after collecting and processing data, produce meaningful feature information and provide a better method to measure ecosystem changes [15]. A research project conducted at the Academia Sinica Biodiversity Research Center [16] has collected and analyzed audio field signals in forests, thereby constructing characteristic sound field training datasets models for forest environments. Different from [16], this presented algorithms used in this study is entirely new approaches of more samples.

Artificial intelligence (AI) techniques have been widely applied in many fields such as image recognition, speech recognition, characteristic signal models, deduction and reasoning, and data mining to solve problems that otherwise are addressed using traditional calculation methods. Implementation challenges include difficult characteristic classification [17]. Nowadays, big data-related applications are a major application of AI for the algorithmic classification of huge amounts of data to identify more practical optimization decision models. Machine learning classification and recognition methods from AI are then applied to obtain optimal prediction performance [18]. Appropriate machine learning techniques can be applied to acoustic datasets to facilitate model training to obtain prediction solutions with optimal adaptive calculations and minimal errors. In the iterative process of machine learning model training, the loss weighting function is minimized to approximate the solution’s optimization trend to train a prediction model that most closely approximates an ideal solution [19, 20]. All in all, this research focuses on the basic application of artificial intelligence through the feature extraction of original signals through filtering calculations, and the classification and recognition of feature spectrum datasets using machine learning techniques.

So-called machine learning (ML) techniques can deduce a system’s optimal model solution from large datasets, and simultaneously perform large volume data analysis and classification. The model is trained from known datasets, and testing data is used to extract the most suitable prediction solution [21]. ML provides complementary data modeling techniques with traditional statistical methods [22]. Among modern algorithms, deep learning (DL) has attracted widespread attention for its ability to train from large datasets [23]. The present research selected characteristic sounds of 35 amphibian species, using a novel digital speech algorithm to perform digital filtering analysis of the sound characteristics. Increasing demand for big data collection and the advancement of computer processing speeds has driven the use of deep learning techniques in practical applications in many fields. In the field of speech recognition, convolutional neural networks (CNN) [24–26], deep neural networks (DNN) [27] long short-term memory (LSTM) [28] and other machine learning methods have been widely used as classification algorithms in recent years. This article introduces deep neural network (DNN) and long short-term memory (LSTM) and discusses to solution of the classification problem for bioacoustic features in practical applications. In bioacoustic digital filtering, both linear predictive coding (LPC) and Mel-frequency cepstral coefficient (MFCC) digital speech algorithms can distinguish characteristic speech signals. These two popular filters are widely used in digital speech signal processing [29, 30], especially in feature extraction of speech signals [31]. The sound feature datasets are used to introduce a mainstream data dimensionality reduction algorithm using principal component analysis (PCA) to perform calculations on a large number of feature datasets, thus reducing dimensionality and calculation loading, thus obtaining better recognition and classification performance. Prior to implementation of image processing or audio feature algorithms, many studies first reduce the dimensionality of big data features to effectively reduce computational complexity and overhead. This PCA method is commonly used for dimensionality reduction in the field of audio signal processing. It helps not only expedite learning efficiency of the datasets but also classify the most effective feature data for further analysis [32].

DNN of the adaptive learning has become major breakthrough in acoustic speech recognition [33, 34]. DNN is a classification algorithm that is often applied to very large amounts of data and is used to develop the proposed experimental framework for bioacoustic classification. The calculation characteristics of the neural network are modulated by a set of digital variables called weights. We seek to optimize the neural network’s calculation performance based on these optimal weights. Based on the multi-layer network connection architecture, we calculate the approximate optimal solution of each node in each neural network. After training a learning model, the neural network is used as an automatic iterative structure to calculate the machine learning training model from the selected input to the required output [35].

In recent years, the long short-term memory (LSTM) algorithm has been increasingly applied for continuous sequential speech signal processing [36, 37]. LSTM is a modified recurrent neural network (RNN) which can store information of previous input for a long time [38]. It can solve the problems of vanishing and exploding gradients along with long sequence training and memory retention [39]. All RNNs have feedback loops in the recurrent layer to help store information in "memory" over time. However, standard RNNs may be difficult to train to resolve the dependence of long-term problems that require learning. The gradient of the loss function decays exponentially over time (a phenomenon called the vanishing gradient problem), making training for a typical RNN difficult. This is why the modified RNN is modified to include a memory cell that can maintain information in memory over time. The most widely used modified RNN is called LSTM, which uses a set of gates to control when information enters the memory, thus solving the vanishing or exploding gradient problem [40]. In this study, animal acoustic features are classified using the Python pytorch platform and we analyze the performance of the two previously mentioned algorithms using principal component analysis in terms of calculation time, and performance. We then filter out the most suitable category recognition algorithm classification structure for this dataset. Later in the article we discuss the influence of principal component analysis on deep neural networks and long and short-term memory, and further infer the respective advantages of the two calculation methods.

## 2. Theoretical description

### 2.1. Linear Predictive Coding (LPC) method

**Fig 1:**
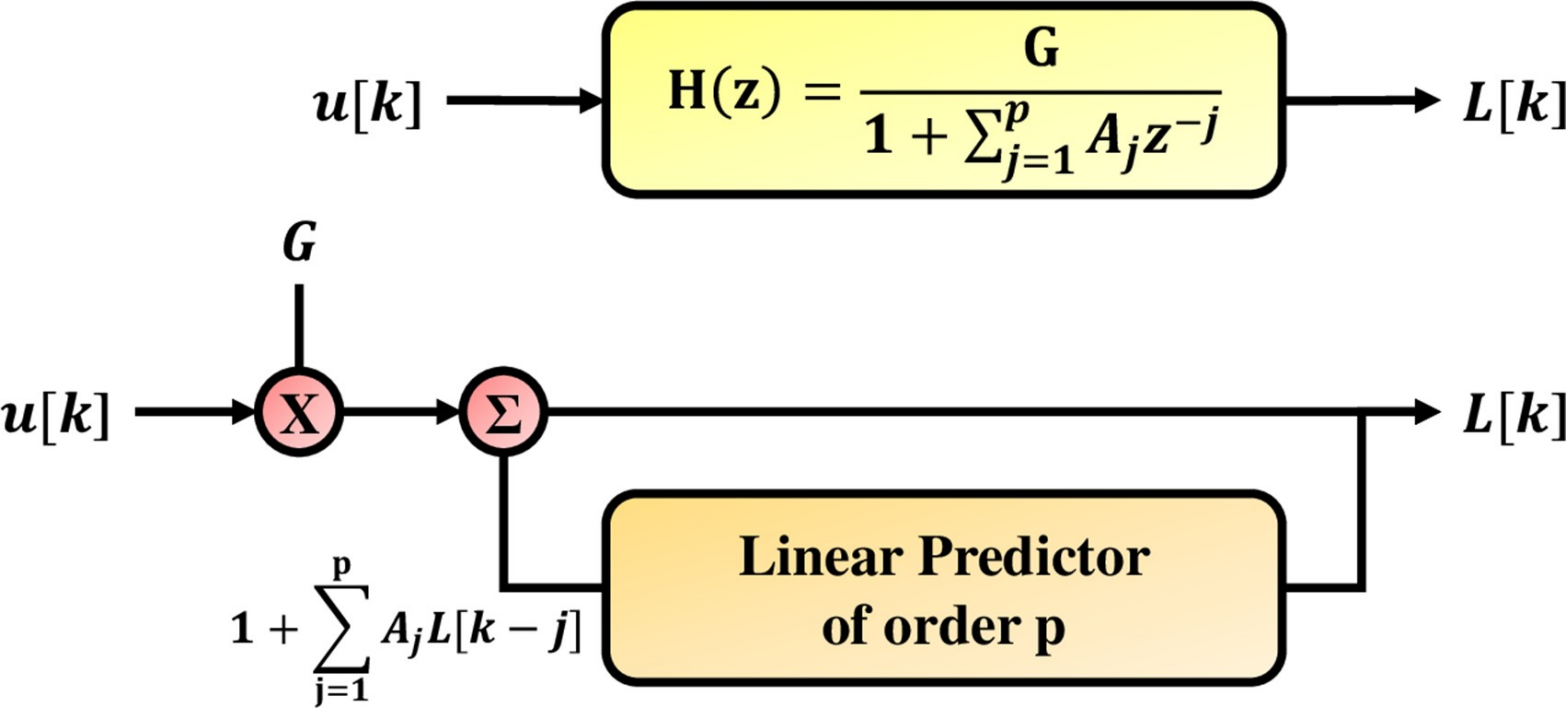
This figure presents the speech production model through LPC method.

The digital speech linear predictive coding (LPC) method describes that a sample *L*[*k*] can be approximately expressed as a function of the linear combination of the previous samples [41], which is $L[k]=\sum_{m=1}^{P}a_{m}L[k-m]$. {*a_m_*} represents the combined coefficient *k* = 1,2,…*P* called the linear prediction coefficient. The basic structure of LPC algorithm model is illustrated as Fig 1.

The characteristics of LPC is a linear combination of this function [42].

$$L[k]=-\sum_{j=1}^{p}A_{j}s[k-j]+G\sum_{l=0}^{q}B_{l}u[k-l],$$ (1)

where *A*_*j*_ and *B*_*l*_ are prediction coefficients. G is the gain value, and *u*[*k*] represents the unknown input signal.

The z transformation signal *T*(*z*) of signal *L*[*k*] is expressed as [43]:

$$T(z)=\sum_{n=-\infty }^{\infty }L[n]z^{-n}.$$ (2)

The transfer function *H*(*z*) is the output of the filter to the input and corresponds to the following items.


$$H(z)=G\frac{\sum_{l=0}^{q}B_{l}z^{-l}}{1+\sum_{j=1}^{p}A_{j}z^{-j}}.$$ (3)

**Fig 2:**
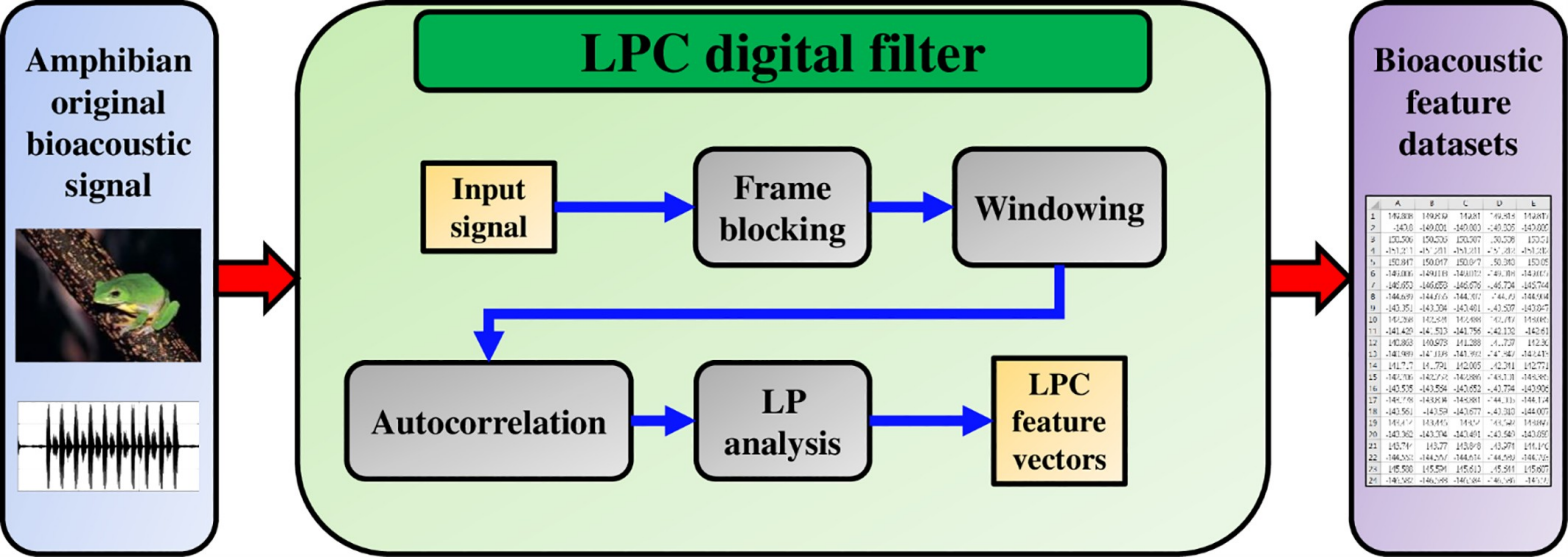
Shows our study based on LPC to construct the bioacoustic feature datasets.

Fig 2 shows the process from collecting the original signals of the amphibian to constructing the bioacoustic feature datasets. With the digital filtering algorithm called LPC, we are able to do feature extraction to the original acoustic signals of every single specy of the amphibian, adjust the linear predictive coefficients to create multiple filtering effects, and collect the feature spectral values of every single specy to construct the training datasets.

### 2.2. Mel-Frequency Cepstral Coefficient (MFCC) method

**Fig 3:**
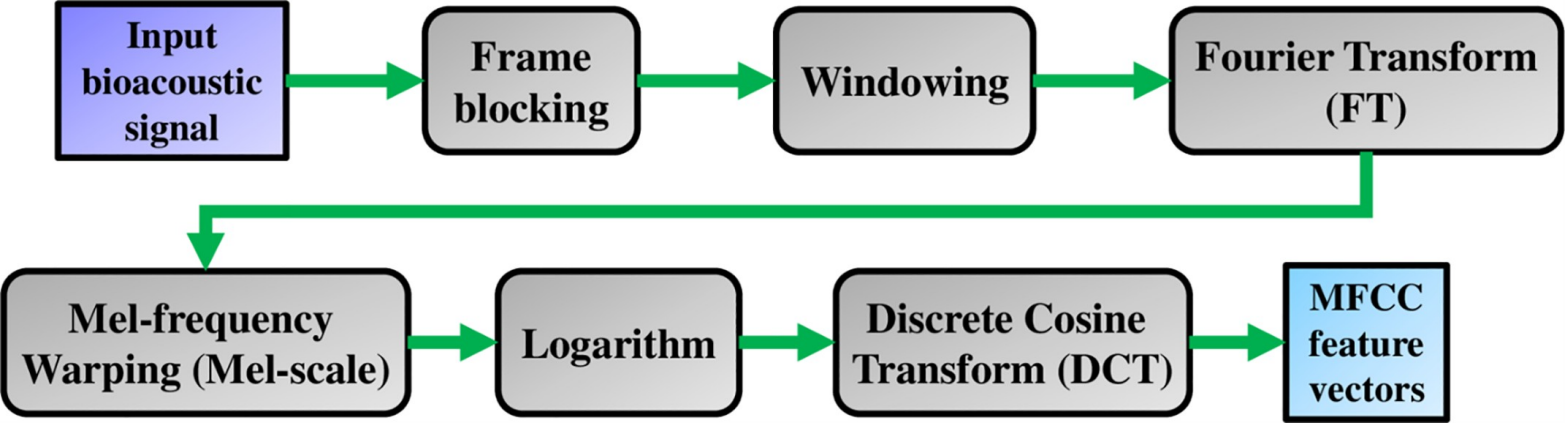
Shows the architecture of the MFCC.

This study is inspired from the feature classification experiments in [16]. The methods in [16] are to use the MFCC digital filtering algorithm to extract features from the original acoustic signals every single specy of the amphibian. The methods in [16] adjust the pre-emphasis coefficients to create multiple filtering effects, collect the feature spectral values, and construct the training datasets. Fig 3 shows the architecture of the MFCC.

### 2.3. Deep Neural Network (DNN) method

DNN provides better feature classification and is suitable for high-complexity mapping. The basic structure of a neural network transforms the input into the desired output that meets the goal. Inputs form input nodes, and outputs are represented as output nodes. The middle layer between the input and output is called the hidden layer. The number of layers is not strictly fixed, and networks typically use more layers. The general function of each neuron in a neural network is basically described as follows [44].


$$y_{j}^{1}=T_{f}(\sum_{i}x_{i}\times w_{ji}^{1}).$$ (4)

**Fig 4:**
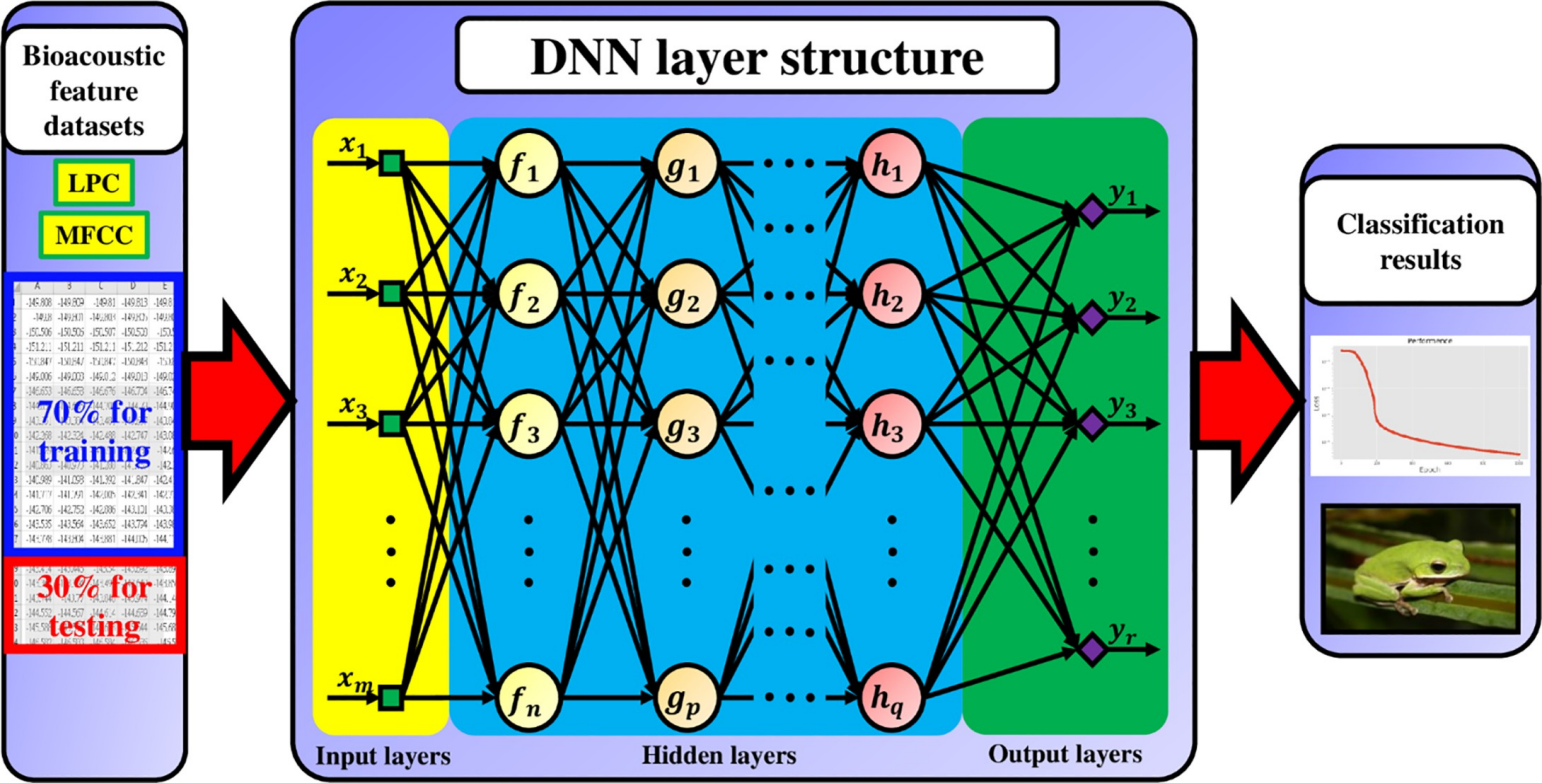
DNN structure consisting of many hidden layers. In the experiment, four structures of DNNs with different hidden layer number are constructed in the classification. There are 10240 feature lengths in the input layer. The output layer generates 35 predictive targets.

In fact, various neural networks can be constructed, depending on how the neurons are connected. Fig 4 shows the constructed datasets based on the digital filter using the first machine learning classifier, DNN, to perform feature classification.

### 2.4. Long Short-Term Memory (LSTM) method

**Fig 5:**
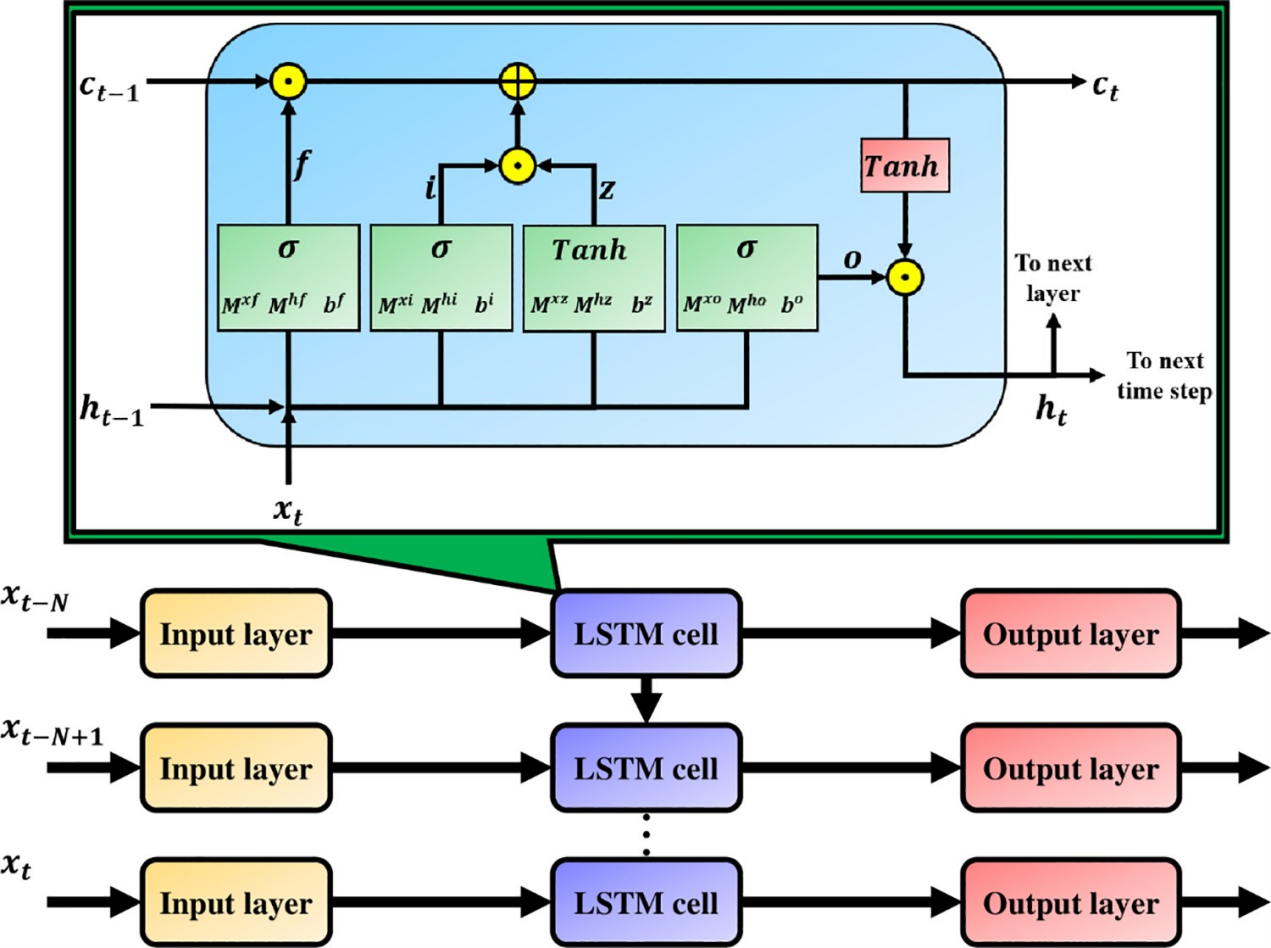
Modern LSTM units and its layer structure are illustrated. Same as those described for Fig 4, there are 10240 feature lengths in the input layer, where he output layer generates 35 predictive targets.

The LSTM architecture is designed to solve the vanishing gradient problem and is the first tool to introduce a gating mechanism. The modern LSTM architecture is shown in Fig 5.

Mathematically, the LSTM structure is defined as [45]:

$$i_{t}=\sigma (M^{xi}x_{t}+{M^{hi}h}_{t-1}+b^{i}),$$ (5)

$$f_{t}=\sigma ({M^{xf}x}_{t}+{M^{hf}h}_{t-1}+b^{f}),$$ (6)

$$z_{t}=\tanh\left(M^{xz}x_{t}+{M^{hz}h}_{t-1}+b^{z}\right),$$ (7)

$$c_{t}=(c_{t-1}⊙f_{t})⊕(i_{t}⊙z_{t}),$$ (8)

$$o_{t}=\sigma (M^{xo}x_{t}+{M^{ho}h}_{t-1}+b^{o}),$$ (9)

$$h_{t}=o_{t}⊙\tanh\left(c_{t}\right),$$ (10)

***i***_***t***_**, *f***_***t***_**, *c***_***t***_ and ***o***_***t***_ are four gates, respectively used for input, forgetting, cell and output. Threshold values are calculated based on the linear combination of the gates, the current input ***x***_***t***_ and the previous state ***h***_***t*−1**_ through the sigmoid activation function. The updated candidate ***z***_***t***_ is calculated by the linear combination of ***x***_***t***_ and ***h***_***t*−1**_, and pass the tanh activation function. The cell state of the previous time period, ***c***_***t*−1**_, will be modified to obtain the cell state of the current time period, ***c***_***t***_, and this process is not directly related to any weight factor multiplication. The output gate determines how to update the values of the hidden units [46]. Similar to the aforementioned DNN method, the training model constructed by the digital filter is introduced in this experiment through the second machine learning classifier using long and short-term memory (LSTM) to perform feature classification.

### 2.5. Principal Component Analysis (PCA) method

The number of so-called principal components is basically less than or equal to the number of original variables. The main concept of this conversion is that the first principal component contains the largest possible variance [43]. The matrix to map the vector *x*_*i*_ in the feature dimension to the corresponding vector *u*_*i*_ in the lower dimension needs to be defined. The set of vectors *y*_*i*_ and *x*_*i*_ corresponds to *y*_*i*_ = *M*^*T*^*x*_*i*_. The scattering matrix calculated in the eigen-dimensional vector can be expressed as [43]:

$$F_{v}=\sum_{i=1}^{i=N}{\left(x_{i}-\mu \right)}^{T}\;(x_{i}-\mu ),$$ (11)

where $\mu =\frac{\sum_{i=1}^{i=N}x_{i}}{N}$ represents the mean vector calculated on the feature dimension. Let the scattering matrix calculated from the low-dimensional vector be calculated as *F*_*u*_, which corresponds to *F*_*v*_ because *F*_*u*_ = *M*^*T*^*F*_*v*_*M*.

The transformation matrix M is optimized to maximize the variance of each element in the transformation vector. $M_{k}^{T}F_{v}M_{k}$ is maximized by the constraint $M_{k}^{T}M_{k}=1$. This can be solved by the Langrangian method given as follows.


$$L(M_{k},\lambda_{k})=M_{k}^{T}F_{v}M_{k}-\lambda_{k}(M_{k}^{T}M_{k}-1).$$ (12)

### 2.6. Optimizer function of neural networks

The Adam algorithm exponentially smoothens a step to combine momentum and update. When the processing forecast of the smoothed value is unrealistically initialized to zero, it directly addresses the trend inherent in exponential smoothness [47]. Let *X*_*t*_ be the exponential average of the t^th^ parameter and set it to *w*_*t*_. This value can be modified by a formula similar to RMSProp, but the parameter is *ρ* and the range is 0 to 1 [47].


$$X_{t}\leftarrow \rho X_{t}+(1-\rho ){\left(\frac{\partial L}{\partial w_{t}}\right)}^{2}\;\forall t.$$ (13)

This gradient is maintained with exponentially smoothed values, for which the t^th^ component is denoted as *F*_*t*_. The smoothing process is also represented by another attenuation parameter *ρ*_*f*_.


$$F_{t}\leftarrow \rho_{f}F_{t}+(1-\rho_{f})\left(\frac{\partial L}{\partial w_{t}}\right)\;\forall t.$$ (14)

Adaptive Moment Estimation optimizer (Adam) is widely used because it combines the advantages of many optimizers and is quite competitive [47]. It is used here as an optimizer function for deep neural networks (DNN) and long short-term memory (LSTM).

## 3. Experimental methods and verification

### 3.1. Raw data information of anuras

**Fig 6:**
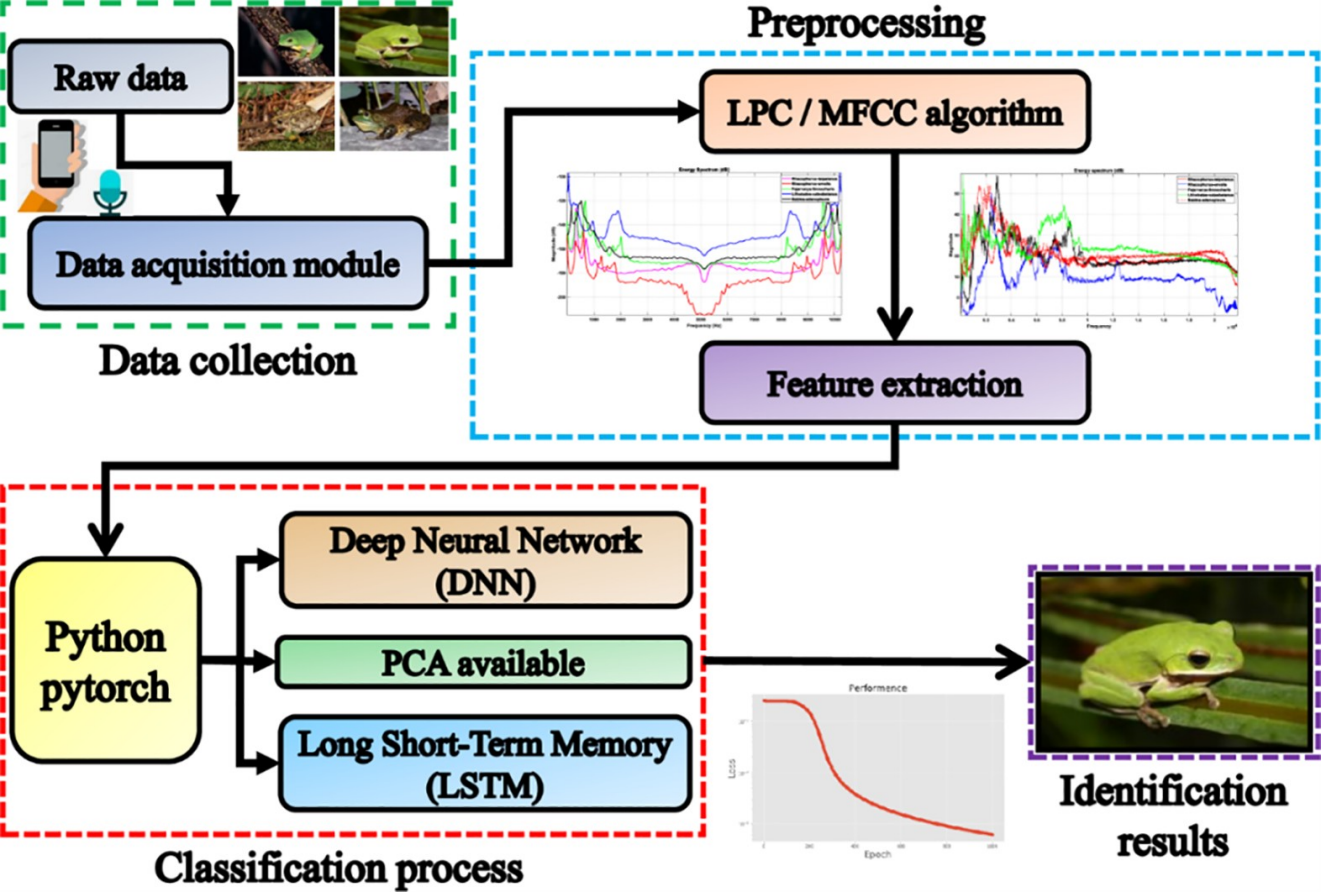
The structure of the experimental process for anuran bioacoustic classification.

**Fig 7:**
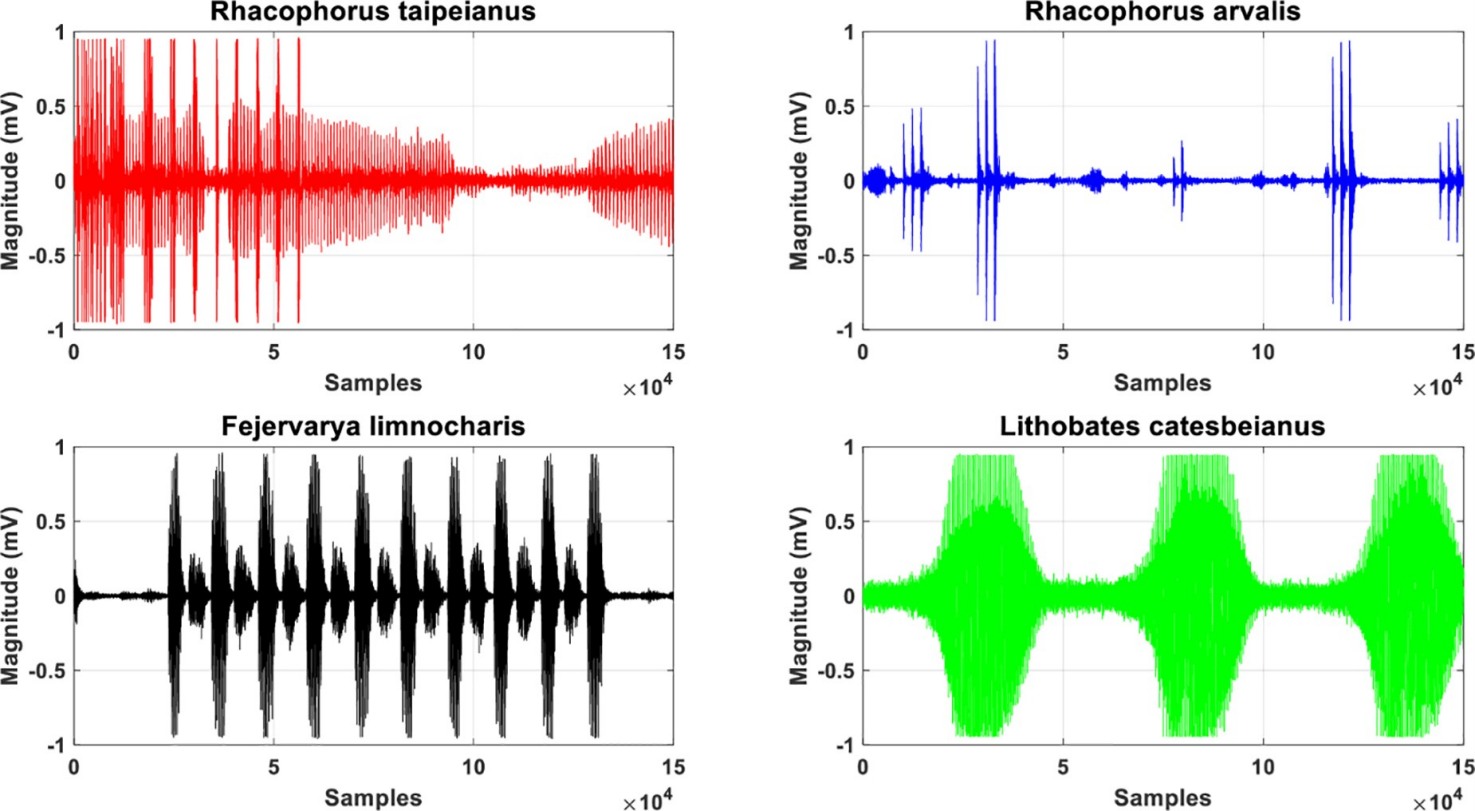
The collected data information of the first 4 anuras, including *Rhacophorus taipeianus*, *Rhacophorus arvalis*, *Fejervarya limnocharis*, *Lithobates catesbeianus*, is plotted with time length of approximately 20 seconds for each raw data.

Roughly speaking, the experiment is divided into four main steps: collection of animals bioacoustic data, characteristic digital speech signal processing, classification, and recognition [48]. Fig 6 shows the experimental structure of the process [16, 49]. Table 1 below lists the 35 amphibians for which bioacoustics were collected. The source of the bioacoustic data sets can be found in http://learning.froghome.org/D/index.html. The signal sampling rate is 44100Hz, and the time series data captured by each sound file is about 20 seconds. Prior to processing, we first obtain the original amphibian audio as shown in Fig 7.

**Table 1:** Anuran species for classification.

Scientific Name of Anuras	Species	Scientific Name of Anuras	Species
*Rhacophorus taipeianus*	Frog	*Kaloula pulchra*	Frog
*Rhacophorus arvalis*	Frog	*Limnonectes fujianensis*	Frog
*Fejervarya limnocharis*	Frog	*Rana latouchii*	Frog
*Lithobates catesbeianus*	Frog	*Fejervarya cancrivora*	Frog
*Babina adenopleura*	Frog	*Buergeria japonica*	Frog
*Microhyla ornata*	Frog	*Buergeria otai*	Frog
*Rana longicrus*	Frog	*Buergeria robusta*	Frog
*Hoplobatrachus rugulosus*	Frog	*Kurixalus eiffingeri*	Frog
*Hylarana taipehensis*	Frog	*Kurixalus idiootocus*	Frog
*Pelophylax plancyi*	Frog	*Polypedates braueri*	Frog
*Polypedates megacephalus*	Frog	*Rhacophorus aurantiventris*	Frog
*Pseudoamolops sauteri*	Frog	*Rhacophorus moltrechti*	Frog
*Odorrana swinhoana*	Frog	*Rhacophorus prasinatus*	Frog
*Rana okinavana*	Frog	*Khirixalus wangi*	Frog
*Rana guentheri*	Frog	*Bufo bankorensis*	Toad
*Microhyla butleri*	Frog	*Duttaphrynus melanostictus*	Toad
*Microhyla heymonsi*	Frog	*Hyla chinensis*	Toad
*Micryletta steinegeri*	Frog		

### 3.2. Bioacoustic filtering processing

**Fig 8:**
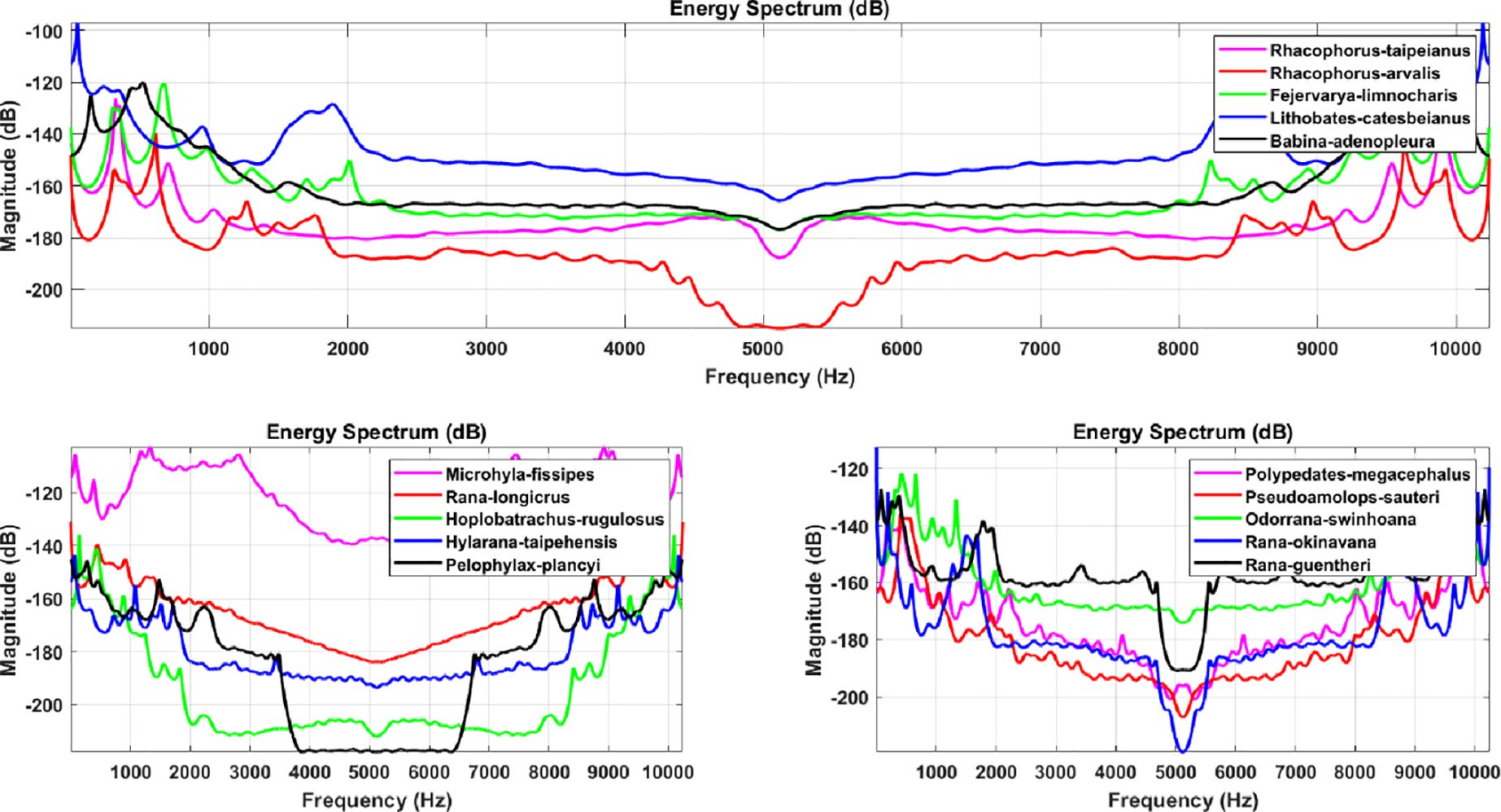
The spectrum diagram of anuran bioacoustic features filtered through the LPC algorithm with P coefficient equal to 60, including *Rhacophorus taipeianus*, *Rhacophorus arvalis*, *Fejervarya limnocharis*, *Lithobates catesbeianus*, *Babina adenopleura*, *Microhyla ornata*, *Rana longicrus*, *Hoplobatrachus rugulosus*, *Hylarana taipehensis*, *Pelophylax plancyi*, *Polypedates megacephalus*, *Pseudoamolops sauteri*, *Odorrana swinhoana*, *Rana okinavana* and *Rana guentheri*.

**Fig 9:**
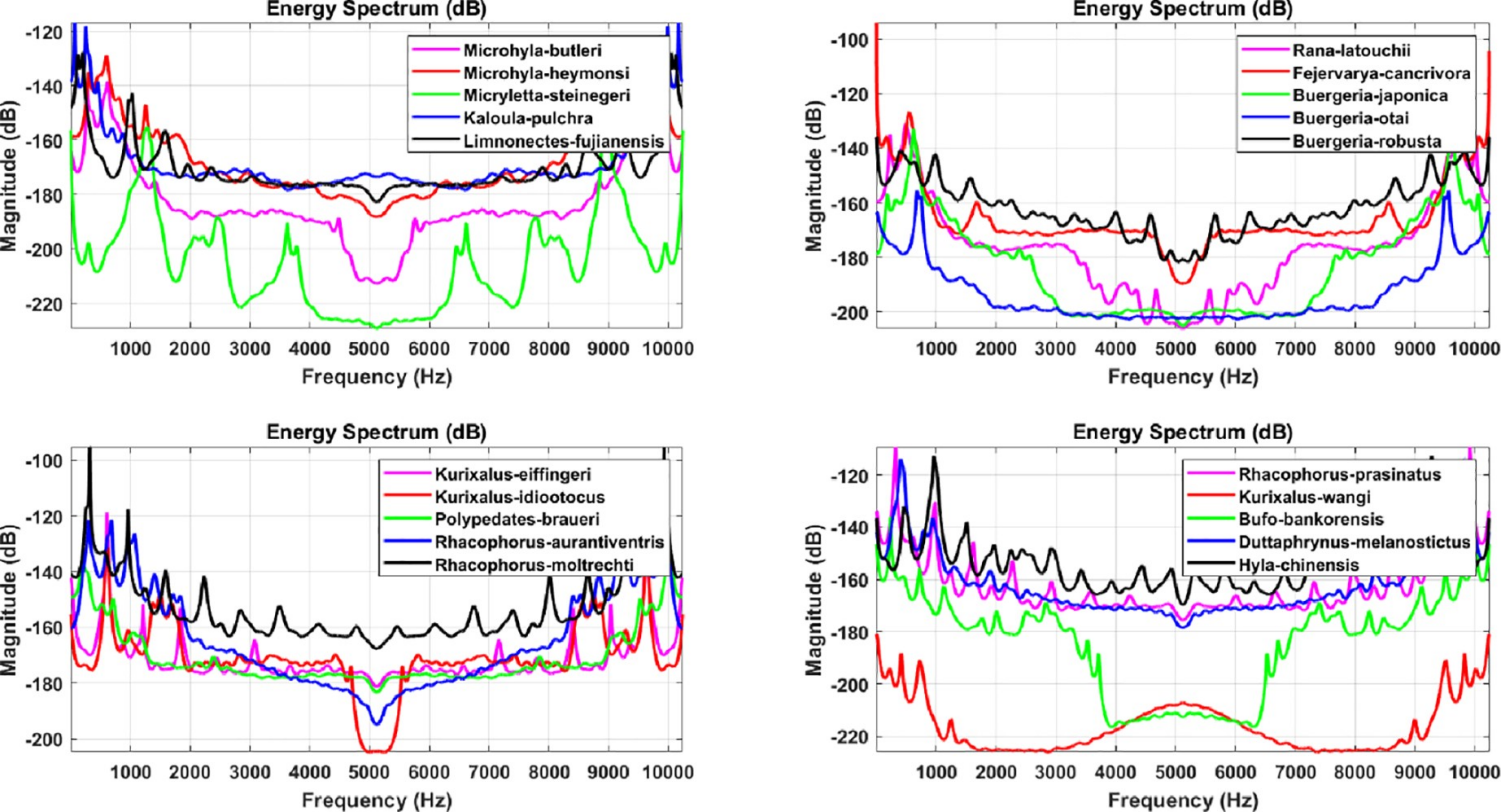
The spectrum diagram of anuran bioacoustic features filtered through the LPC algorithm with P coefficient equal to 60, including *Microhyla butleri*, *Microhyla heymonsi*, *Micryletta steinegeri*, *Kaloula pulchra*, *Limnonectes fujianensis*, *Rana latouchii*, *Fejervarya cancrivora*, *Buergeria japonica*, *Buergeria otai*, *Buergeria robusta*, *Kurixalus eiffingeri*, *Kurixalus idiootocus*, *Polypedates braueri*, *Rhacophorus aurantiventris*, *Rhacophorus moltrechti*, *Rhacophorus prasinatus*, *Khirixalus wangi*, *Bufo bankorensis*, *Duttaphrynus melanostictus* and *Hyla chinensis*.

**Fig 10:**
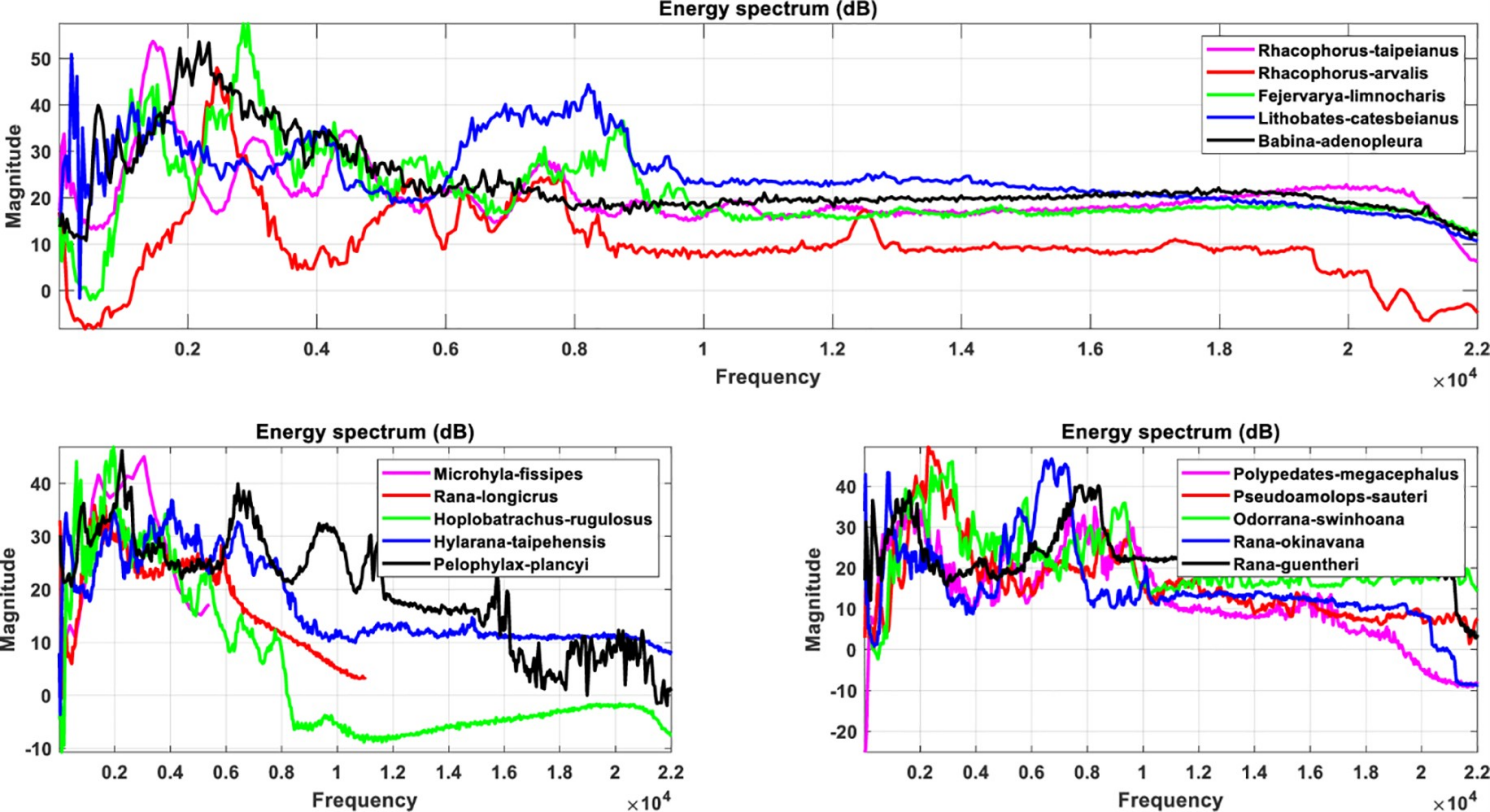
The spectrum diagram of anuran bioacoustic features filtered through the MFCC algorithm with pre-emphasis coefficient equal to 0.9, including *Rhacophorus taipeianus*, *Rhacophorus arvalis*, *Fejervarya limnocharis*, *Lithobates catesbeianus*, *Babina adenopleura*, *Microhyla ornata*, *Rana longicrus*, *Hoplobatrachus rugulosus*, *Hylarana taipehensis*, *Pelophylax plancyi*, *Polypedates megacephalus*, *Pseudoamolops sauteri*, *Odorrana swinhoana*, *Rana okinavana* and *Rana guentheri*.

**Fig 11:**
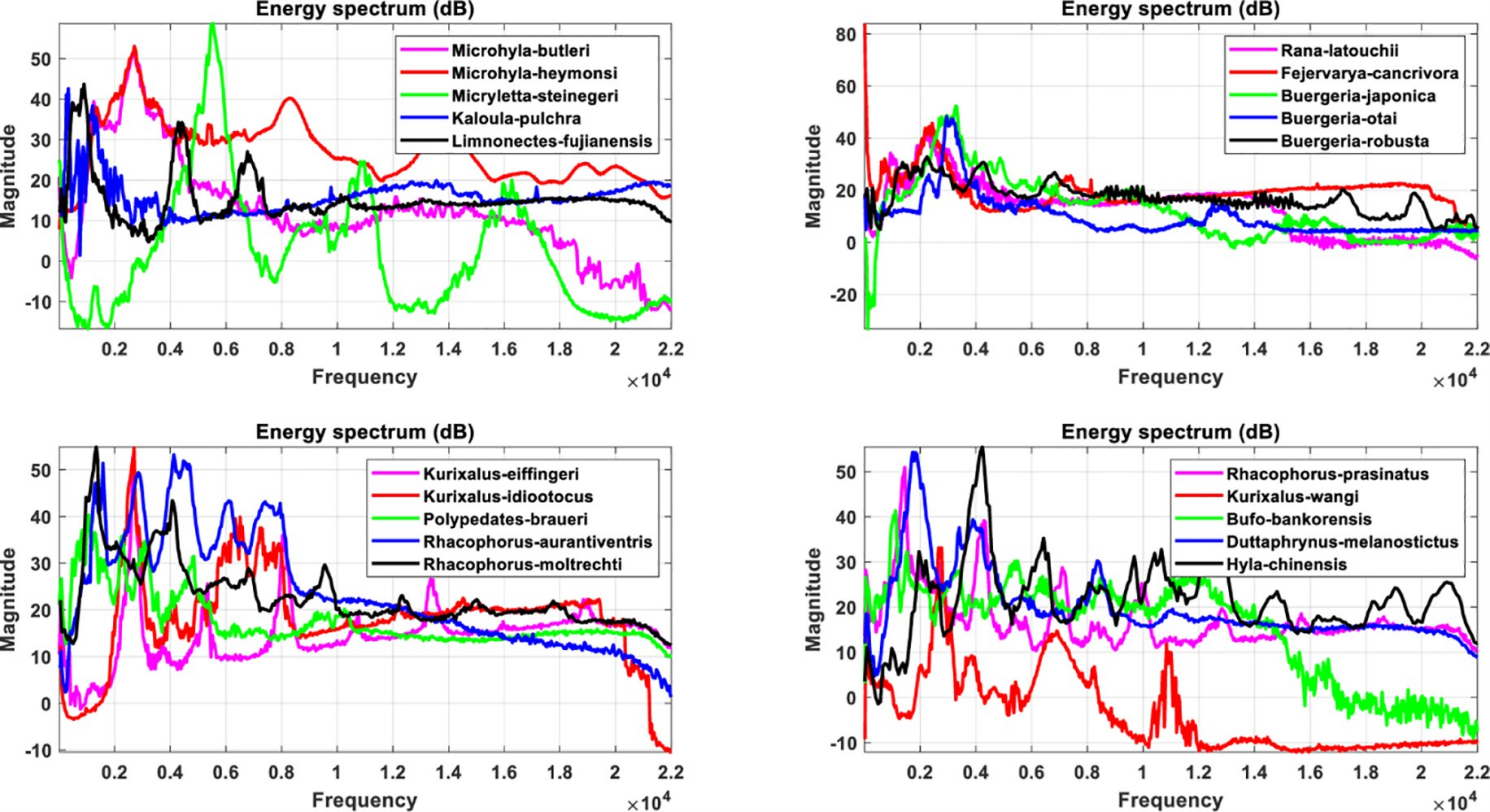
The spectrum diagram of anuran bioacoustic features filtered through the MFCC algorithm with pre-emphasis coefficient equal to 0.9, including *Microhyla butleri*, *Microhyla heymonsi*, *Micryletta steinegeri*, *Kaloula pulchra*, *Limnonectes fujianensis*, *Rana latouchii*, *Fejervarya cancrivora*, *Buergeria japonica*, *Buergeria otai*, *Buergeria robusta*, *Kurixalus eiffingeri*, *Kurixalus idiootocus*, *Polypedates braueri*, *Rhacophorus aurantiventris*, *Rhacophorus moltrechti*, *Rhacophorus prasinatus*, *Khirixalus wangi*, *Bufo bankorensis*, *Duttaphrynus melanostictus* and *Hyla chinensis*.

**Fig 12:**
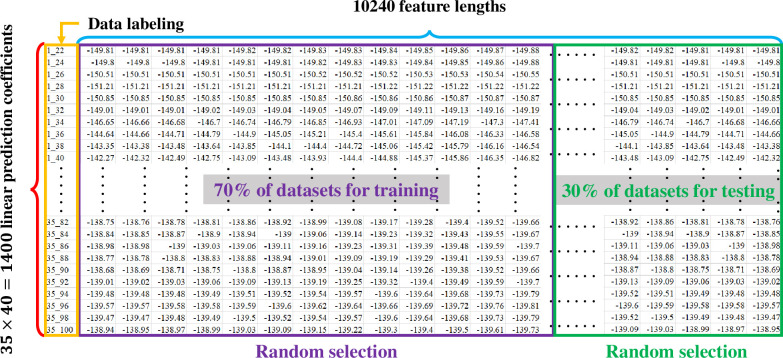
The label establishment of 35 anuran datasets through bioacoustic spectral features filtered by an LPC algorithm. The label in the first column, X_YY, indicates the X-th anura with linear prediction coefficient equal to YY. MFCC also uses similar data labeling and data model construction methods to generate 10240 feature lengths corresponding to the 40 pre-emphasis coefficients. The two datasets are divided into two parts in the machine learning classification stage. The experiment randomly selects 70% of the datasets for training, with the remaining 30% used for testing.

The LPC as well as MFCC filtering algorithms convert the signal from a common timing signal to a bioacoustic spectrum feature, as shown in Figs 8 and 9 for LPC and Figs 10 and 11 for MFCC. First of all, the construction of the feature data datasets is based on 35 types of amphibians, each with 40 sets of LPC coefficients. The P value of the linear estimation filter ranges from 22 to 100 and obtains one every 2 intervals, so there are a total of 1400 feature spectral coefficients. The number of feature lengths selected for each coefficient is 10240, so the experimental feature spectrum datasets are in the form of a 1400×10240 matrix as shown in Fig 12, which belongs to multi-label multi-class datasets. In the same way, the MFCC method uses 40 pre-emphasis coefficients for each of 35 categories to construct feature datasets. The selection range of the pre-emphasis coefficients ranges from 0.22 to 1 with an interval of 0.02. There are also 1400 feature spectral coefficients, each with a feature length of 10240.

### 3.3. Results of classification and identification

In terms of category recognition applications, the DNN and LSTM are used for feature recognition in this experiment to train bioacoustic feature datasets. Pytorch is a very popular computing platform that uses a parallel decentralized calculation GPU processor for feature data classification using the “Adam” as the optimizer function. In the experimental process, a PCA classification method that can be used for dimensionality reduction of sound spectrum datasets is used out to compare the effectiveness of each algorithm’s architecture, where the number of principal component has been set as 200.

There are four important parameter settings: the number of iterations is set to 1000, the learning rate is set to 0.00002, and batch size is set to 1400, which means that the training process for this model is an iterative operation to calculate neural network weighting and update the value. The ratio of randomly selected validation datasets is 0.3, which means that 30% of the model datasets are randomly selected as testing datasets, which is the basis for model calculation verification. Moreover, LPC and MFCC perform feature classification based on the two deep learning classifiers mentioned previously.

The first classifier used in this study is deep neural network. We construct four different DNN models for classification during the classifying process. Table 2 shows the four types of deep neural network models. Model 1 through 4 respectively have 12, 16, 20 and 24 hidden layers. The activation function used in every neural network here is sigmoid activation function, where the number of inputs here is 10240 feature lengths. The output layer has predicted target number of 35.

**Table 2:** Deep neural network models with labels.

DNN models
12-layer structure: [50,80,100,120,180,200,200,180,120,100,80,50]
16-layer structure: [50,80,100,120,180,200,240,300,300,240,200,180,120,100,80,50]
20-layer structure: [50,80,100,120,180,200,240,300,320,360,360,320,300,240,200,180,120,100,80,50]
24-layer structure: [50,80,100,120,180,200,240,300,320,360,400,480,480,400,360,320,300,240,200,180,120,100,80,50]

**Fig 13:**
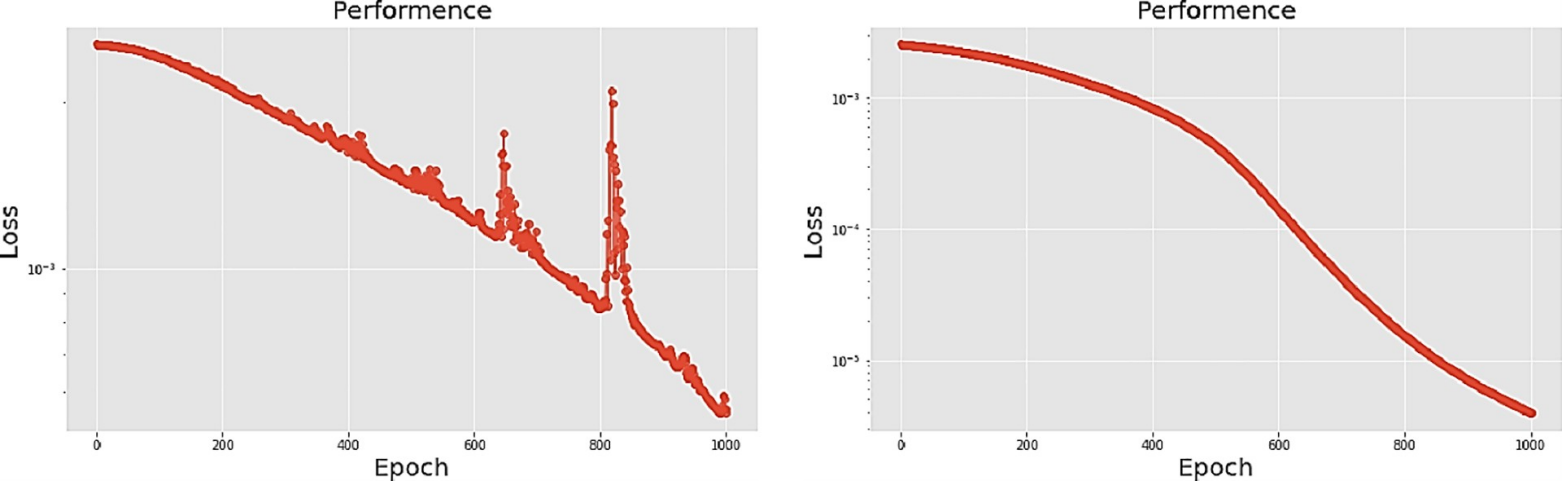
(a) The graph shows the performance of LPC-DNN-12-layer model; (b) The graph shows the performance of LPC-PCA-DNN-12-layer model.

**Fig 14:**
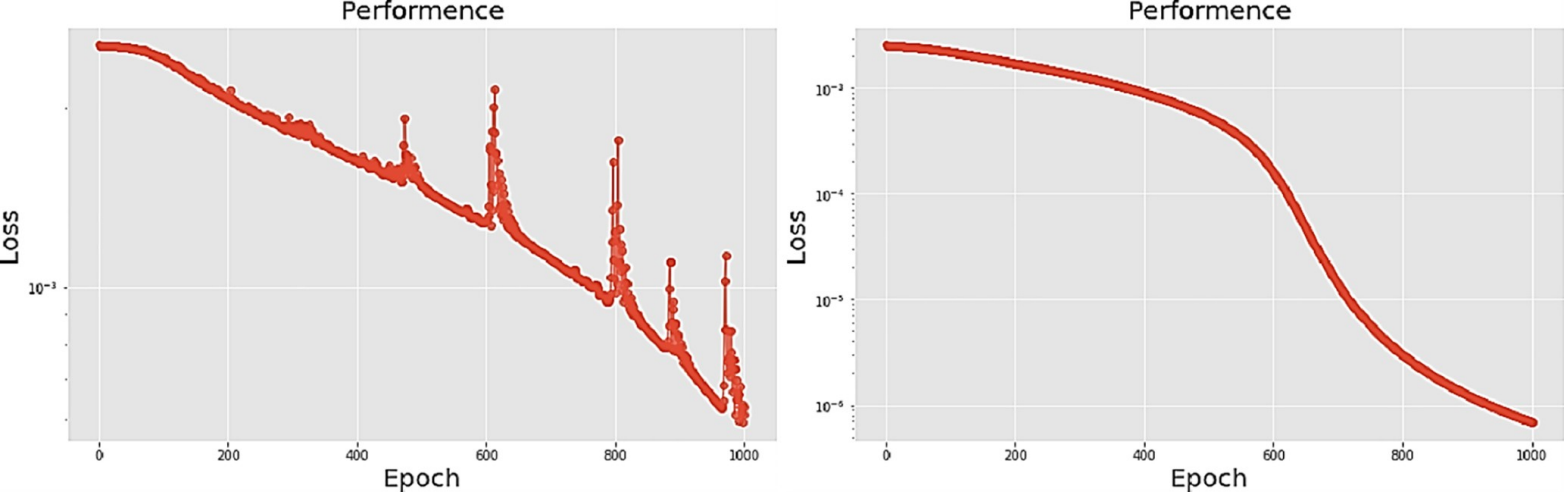
(a) The graph shows the performance of LPC-DNN-16-layer model; (b) The graph shows the performance of LPC-PCA-DNN-16-layer model.

**Fig 15:**
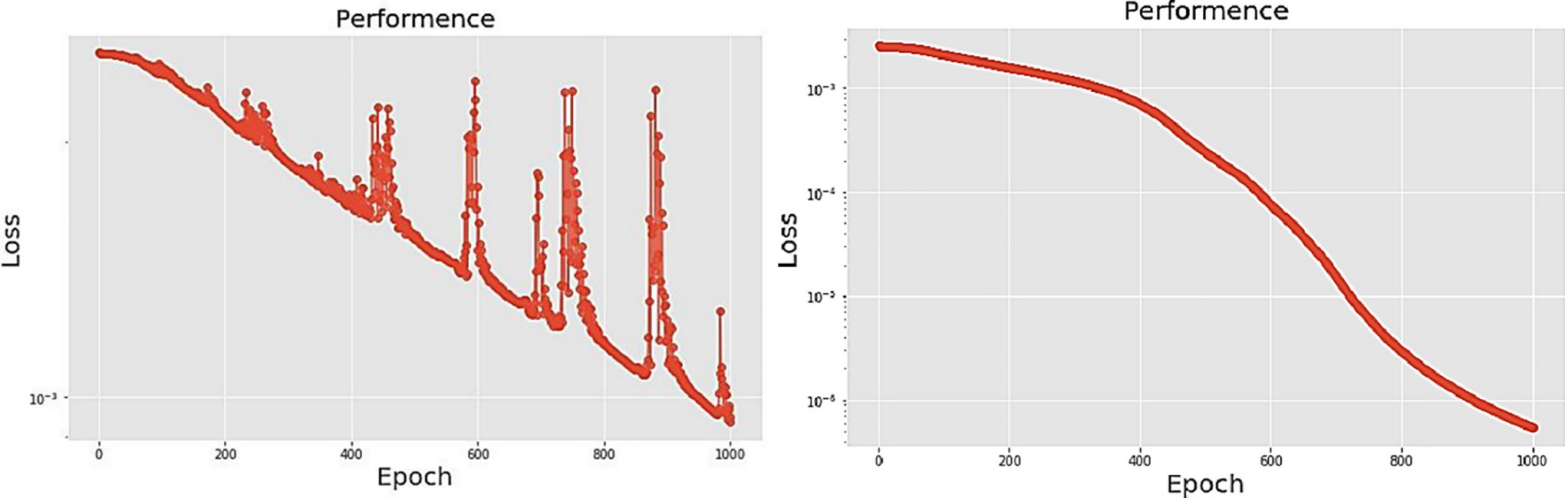
(a) The graph shows the performance of LPC-DNN-20-layer model; (b) The graph shows the performance of LPC-PCA-DNN-20-layer model.

**Fig 16:**
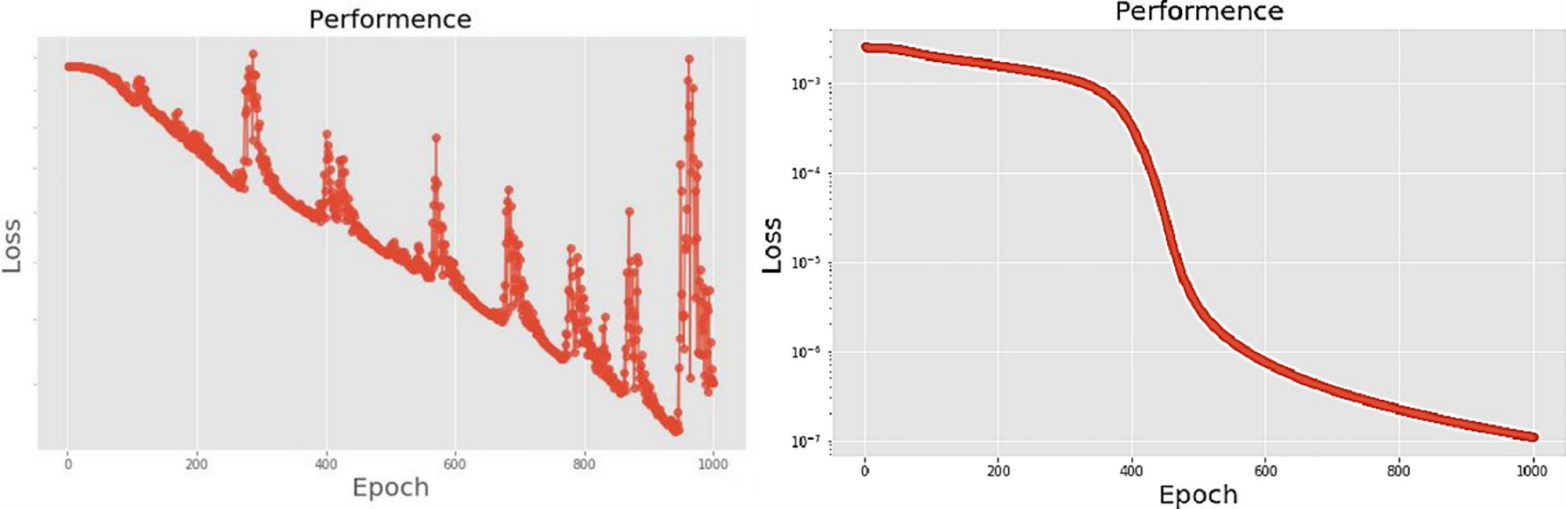
(a) The graph shows the performance of LPC-DNN-24-layer model; (b) The graph shows the performance of LPC-PCA-DNN-24-layer model.

**Fig 17:**
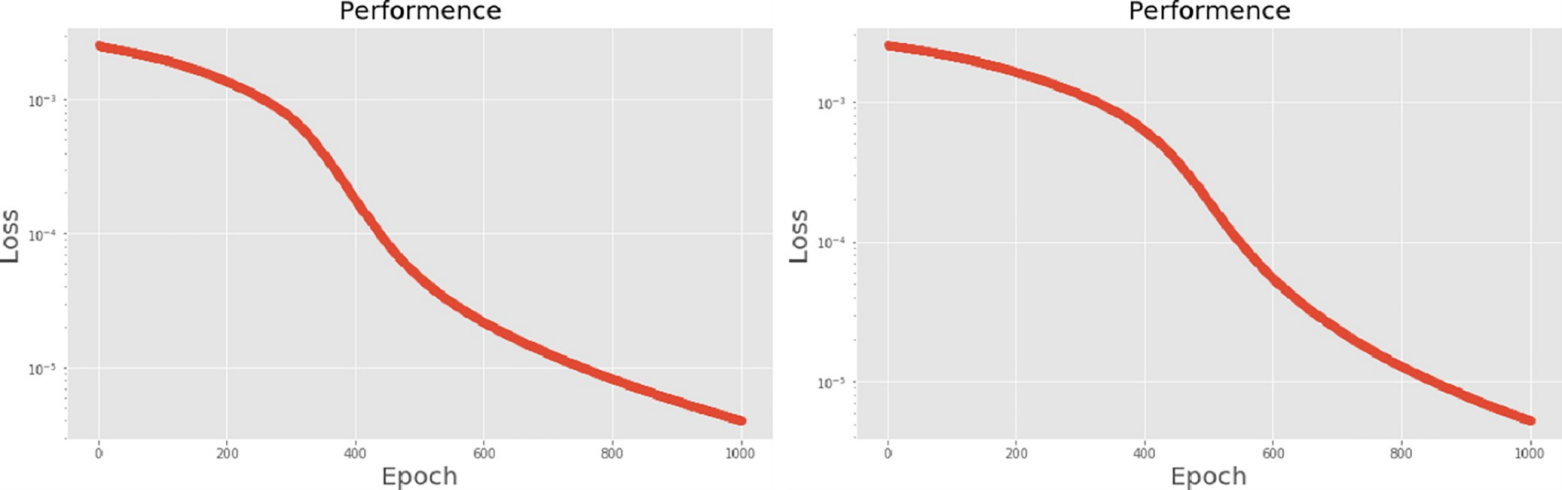
(a) The graph shows the performance of MFCC-DNN-12-layer model; (b) The graph shows the performance of MFCC-PCA-DNN-12-layer model.

**Fig 18:**
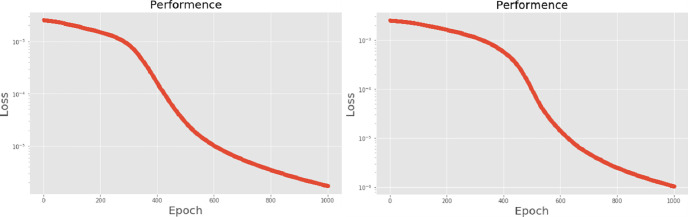
(a) The graph shows the performance of MFCC-DNN-16-layer model; (b) The graph shows the performance of MFCC-PCA-DNN-16-layer model.

**Fig 19:**
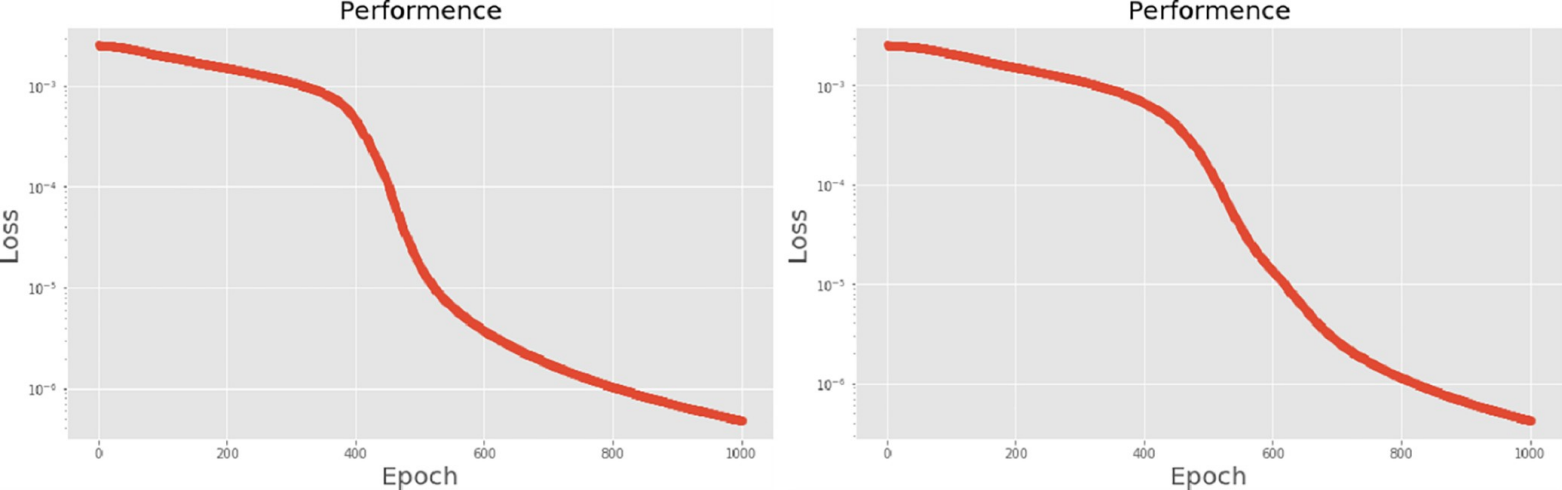
(a) The graph shows the performance of MFCC-DNN-20-layer model; (b) The graph shows the performance of MFCC-PCA-DNN-20-layer model.

**Fig 20:**
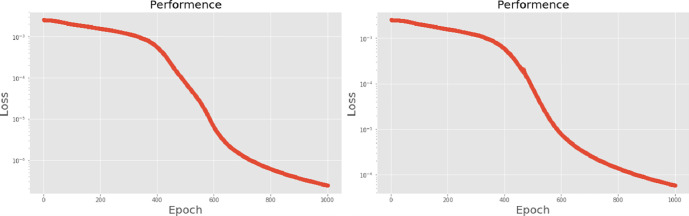
(a) The graph shows the performance of MFCC-DNN-24-layer model; (b) The graph shows the performance of MFCC-PCA-DNN-24-layer model.

Table 3 shows the LPC and MFCC feature classification results of DNN structures from Table 2. For LPC datasets, using PCA for classification increases accuracy while reducing the training period. Figs 13(A), 14(A), 15(A) and 16(A) respectively show the loss function of the LPC-DNN-12-layer, LPC-DNN-16-layer, LPC-DNN-20-layer and LPC-DNN-24-layer models while Figs 13(B), 14(B), 15(B) and 16(B) show the classification process following PCA. Similarly, Figs 17–20 respectively show the similar illustrations as Figs 13–16 but with MFCC filtering algorithm. The LPC and MFCC feature datasets obtain different feature classification results. Compared with the LPC-DNN model, the MFCC-DNN model presents a smoother gradient decent. Introducing the PCA dimensionality reduction method smoothes the gradient descent for both the LPC-PCA-DNN and MFCC-PCA-DNN models. However, the accuracy score calculated by the MFCC-PCA-DNN model is slightly lower than that of the MFCC-DNN model. The performance decline of the model from 12-layers to 24-layers is -0.3%, -0.1%, -0.2% and -1.2% in sequence. This result shows that importing the PCA method has no obvious benefit to the MFCC feature datasets. In addition, as the number of hidden layers of the DNN increases, the accuracy score of the LPC feature datasets is reduced, while the MFCC accuracy remains relatively stable. It can be seen that increasing the number of hidden layers has a greater impact on the LPC model than the MFCC model.

**Table 3:** Training results of DNN models and PCA-DNN models.

**Training model**	**LPC-DNN**	**LPC-PCA-DNN**	**MFCC-DNN**	**MFCC-PCA-DNN**
	**12-layer**	**12-layer**	**12-layer**	**12-layer**
Accuracy score	0.911	1.000	0.991	0.988
Accuracy difference ratio	9.7%		-0.3%	
Training period (sec.)	35.795	34.598	37.052	35.864
Training period difference ratio	-3.3%		-3.2%	
**Training model**	**LPC-DNN**	**LPC-PCA-DNN**	**MFCC-DNN**	**MFCC-PCA-DNN**
	**16-layer**	**16-layer**	**16-layer**	**16-layer**
Accuracy score	0.871	1.000	0.991	0.990
Accuracy difference ratio	14.8%		-0.1%	
Training period (sec.)	40.442	38.329	40.896	42.501
Training period difference ratio	-5.2%		3.9%	
**Training model**	**LPC-DNN**	**LPC-PCA-DNN**	**MFCC-DNN**	**MFCC-PCA-DNN**
	**20-layer**	**20-layer**	**20-layer**	**20-layer**
Accuracy score	0.711	1.000	0.997	0.995
Accuracy difference ratio	40.6%		-0.2%	
Training period (sec.)	44.107	43.765	45.846	43.635
Training period difference ratio	-0.8%		-4.8%	
**Training model**	**LPC-DNN**	**LPC-PCA-DNN**	**MFCC-DNN**	**MFCC-PCA-DNN**
	**24-layer**	**24-layer**	**24-layer**	**24-layer**
Accuracy score	0.454	1.000	0.997	0.986
Accuracy difference ratio	120.1%		-1.2%	
Training period (sec.)	49.533	47.487	48.248	48.822
Training period difference ratio	-4.1%		1.2%	

Nevertheless, sometimes it is not necessary to expand the redundant hidden layers in a DNN, which means that datasets of different sizes will experimentally have the best parameter sets and appropriate structural applications. The impact of PCA implementation on classification effectiveness is clearly revealed in the test results. For the LPC Feature datasets, applying PCA not only reduces the time needed for model training, but also increases the smoothness of the loss function performance. This is counterproductive for the MFCC feature datasets. Moreover, for an appropriate range of neural network structures, classification effectiveness increases with the number of hidden layers.

**Fig 21:**
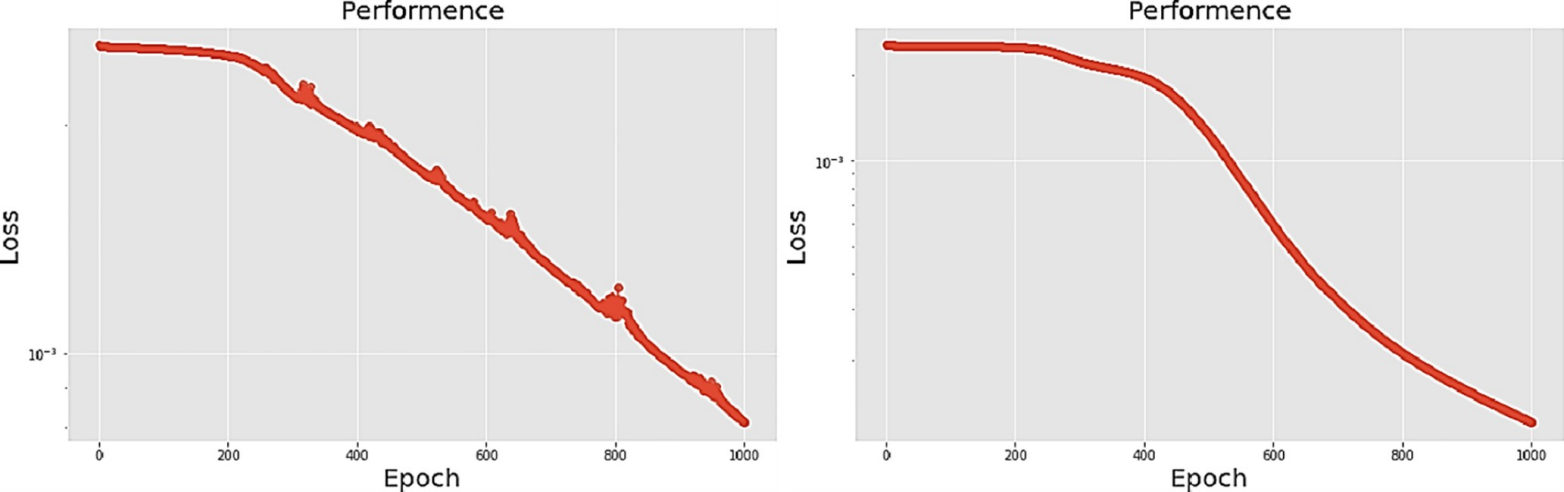
(a) The graph shows the performance of LPC-LSTM-2×200 model; (b) The graph shows the performance of LPC-PCA-LSTM-2×200 model.

**Fig 22:**
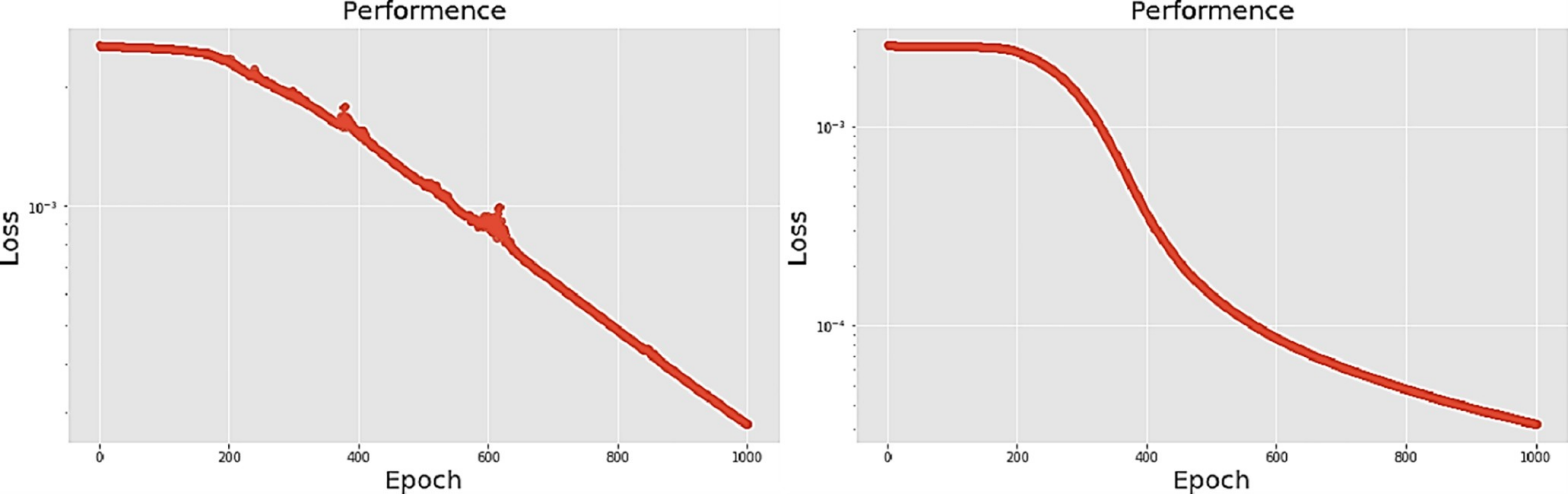
(a) The graph shows the performance of LPC-LSTM-2×300 model; (b) The graph shows the performance of LPC-PCA-LSTM-2×300 model.

**Fig 23:**
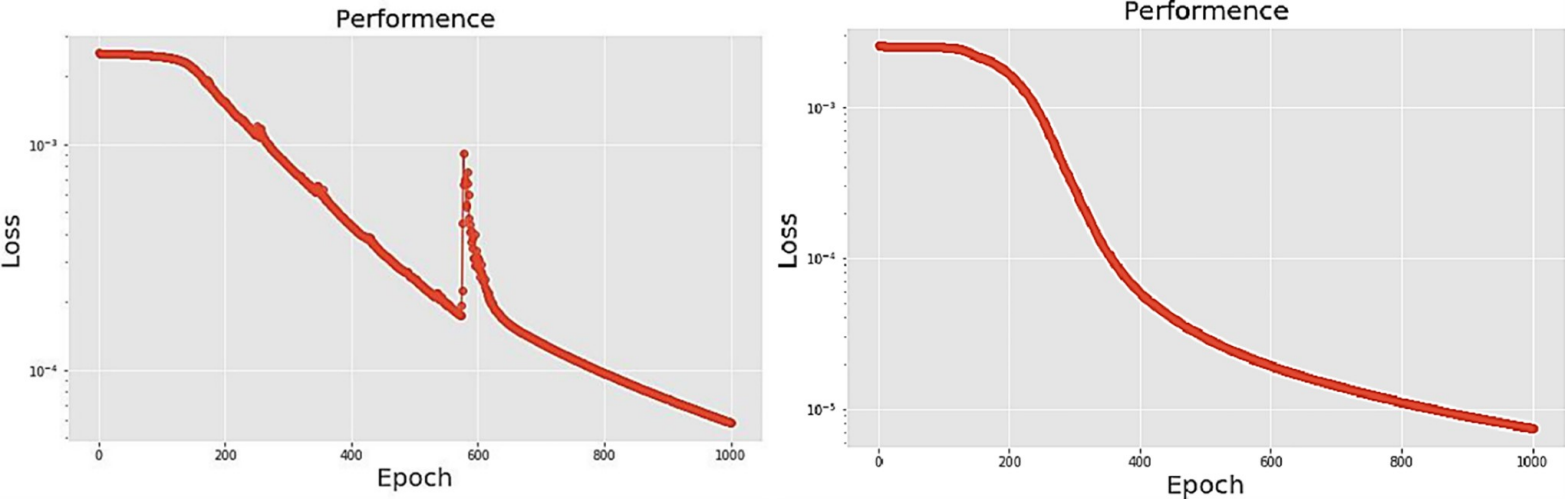
(a) The graph shows the performance of LPC-LSTM-2×500 model; (b) The graph shows the performance of LPC-PCA-LSTM-2×500 model.

**Fig 24:**
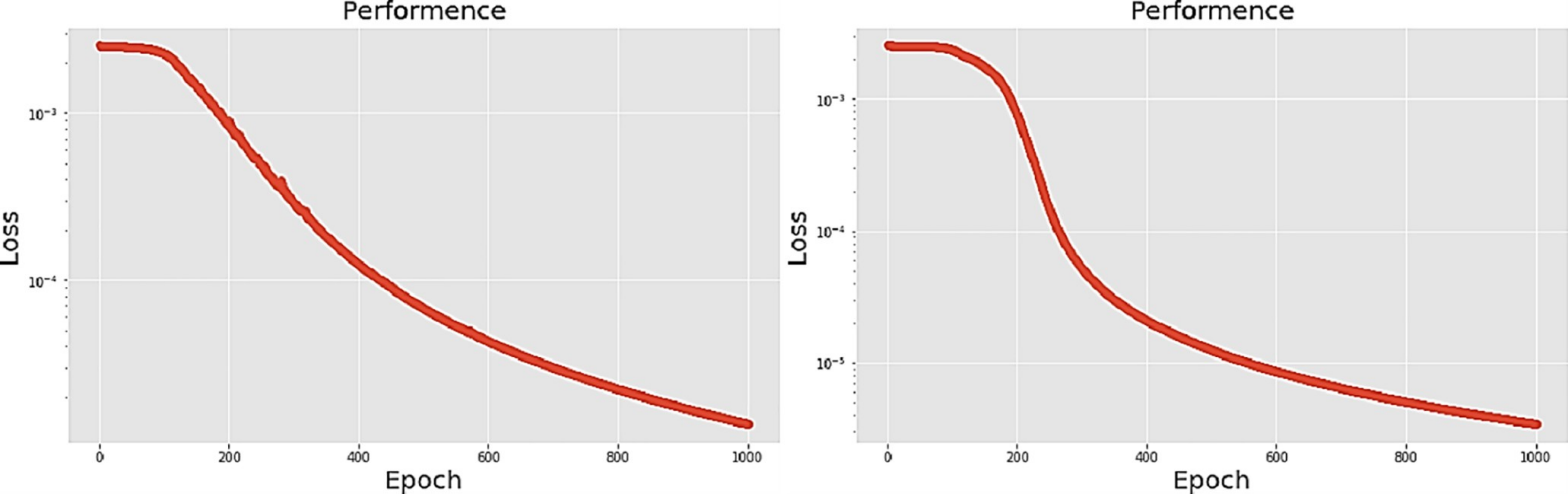
(a) The graph shows the performance of LPC-LSTM-2×700 model; (b) The graph shows the performance of LPC-PCA-LSTM-2×700 model.

**Fig 25:**
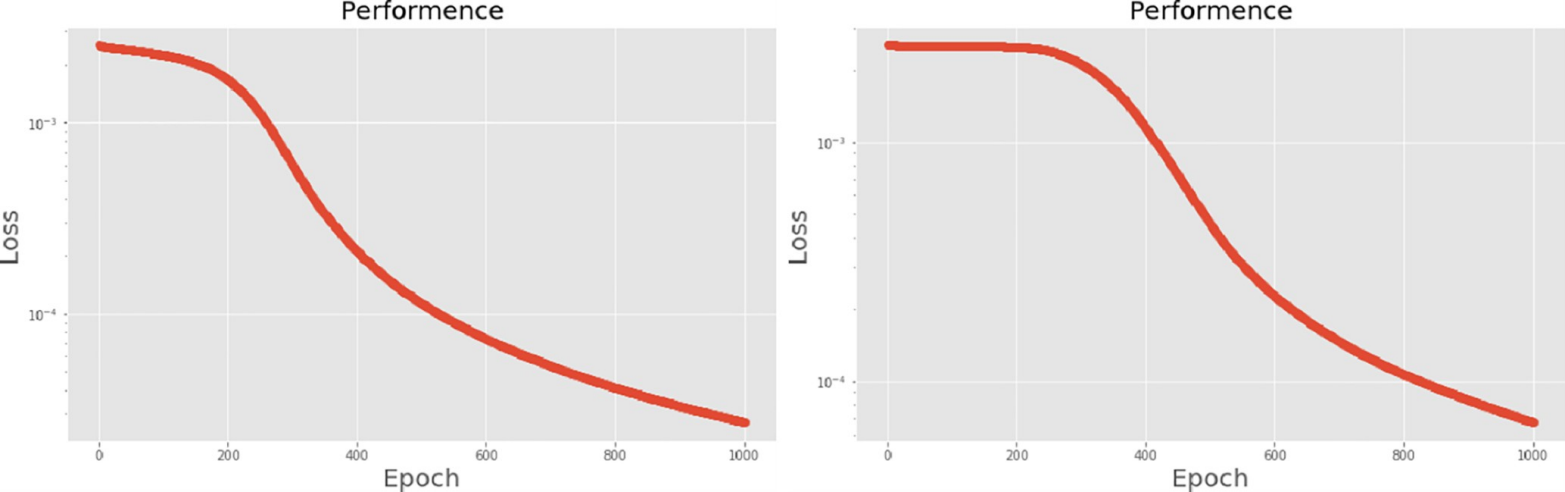
(a) The graph shows the performance of MFCC-LSTM-2×200 model; (b) The graph shows the performance of MFCC-PCA-LSTM-2×200 model.

**Fig 26:**
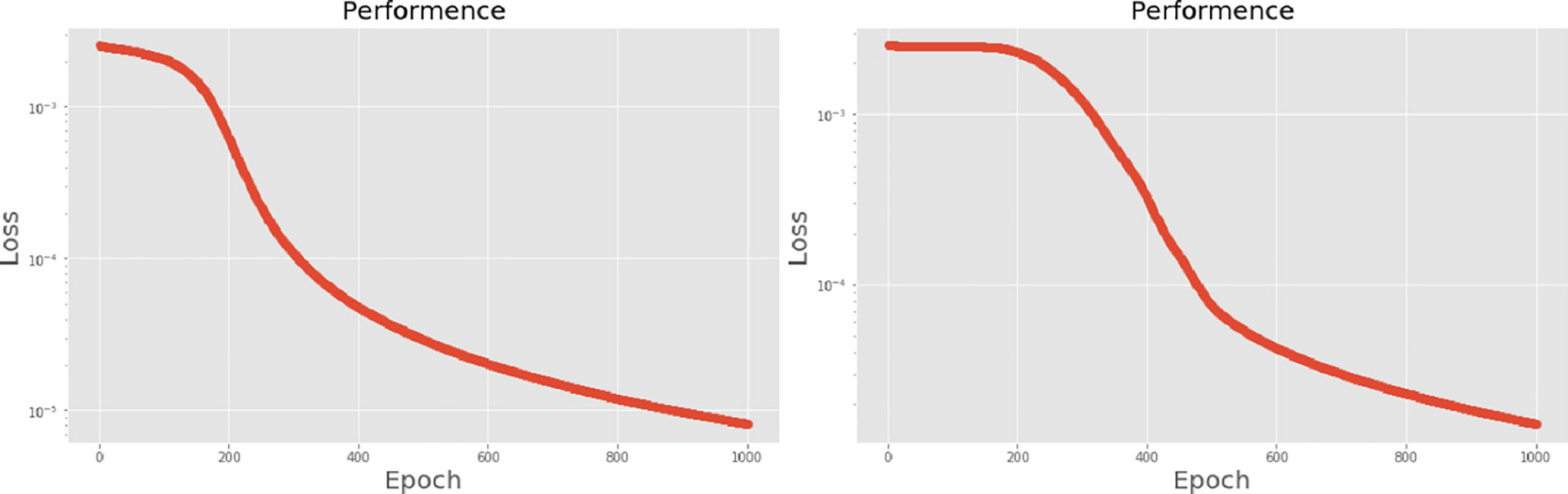
(a) The graph shows the performance of MFCC-LSTM-2×300 model; (b) The graph shows the performance of MFCC-PCA-LSTM-2×300 model.

**Fig 27:**
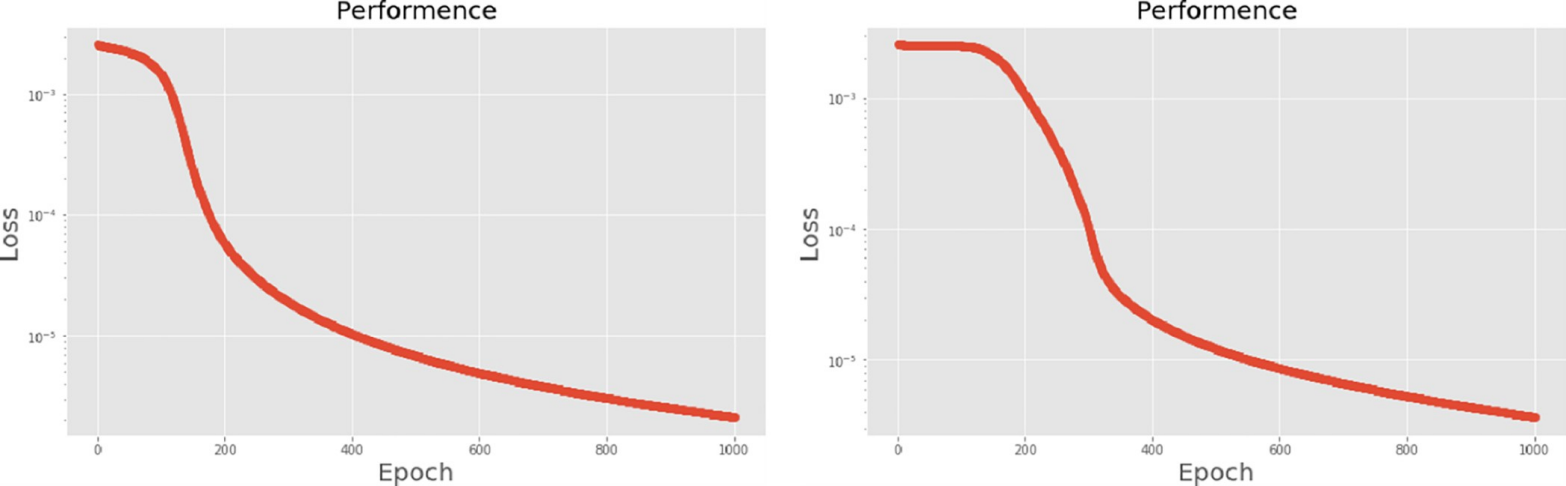
(a) The graph shows the performance of MFCC-LSTM-2×500 model; (b) The graph shows the performance of MFCC-PCA-LSTM-2×500 model.

**Fig 28:**
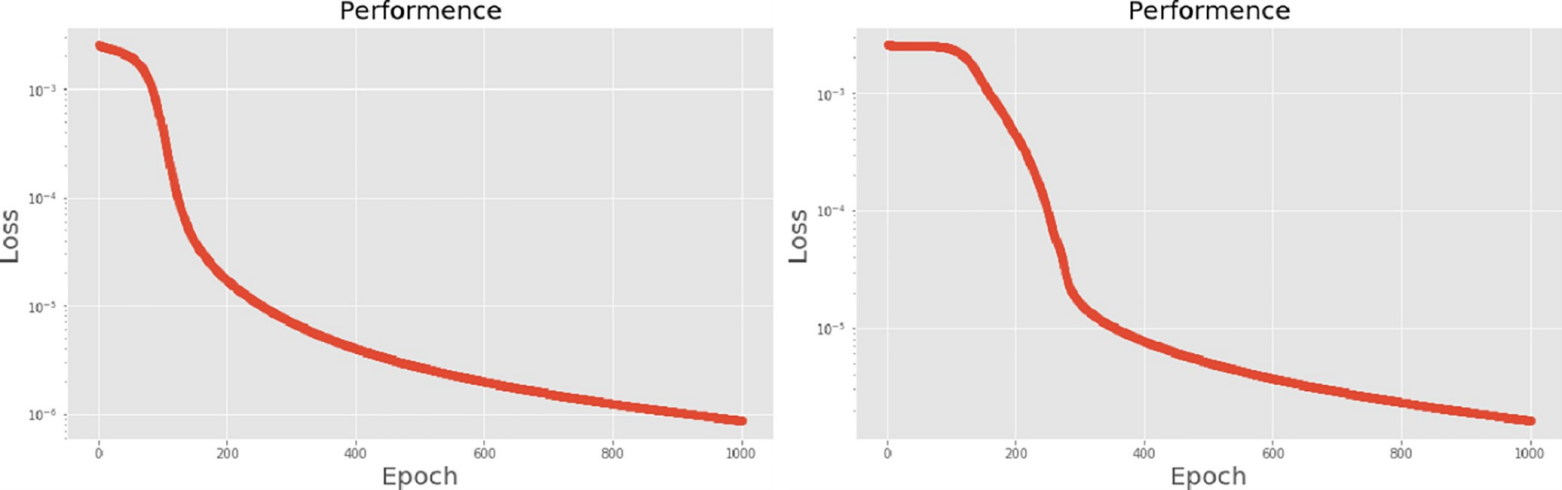
(a) The graph shows the performance of MFCC-LSTM-2×700 model; (b) The graph shows the performance of MFCC-PCA-LSTM-2×700 model.

**Fig 29:**
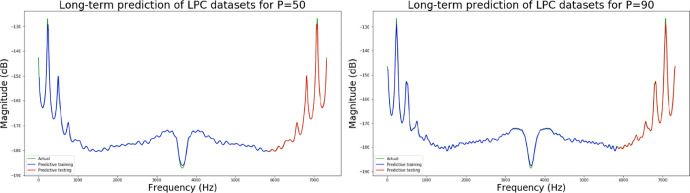
(a) It shows the predictive coefficient in the LPC feature datasets is 50 of the long-term prediction; (b) It shows the predictive coefficient in the LPC feature datasets is 90 of the long-term prediction.

**Fig 30:**
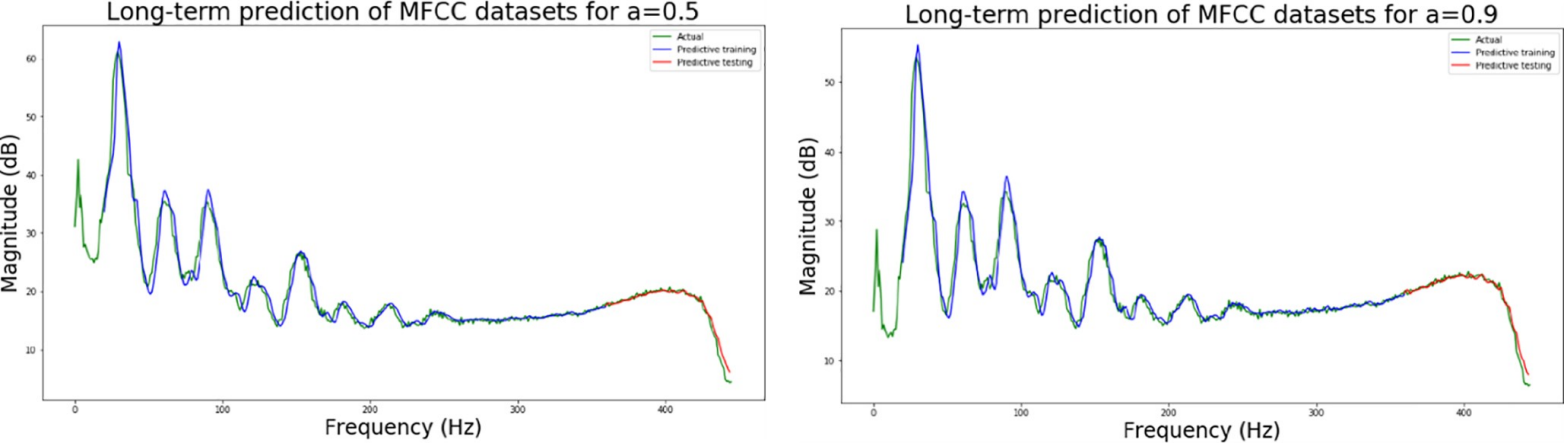
(a) It shows the pre-emphasis coefficient in the MFCC feature datasets is 0.5 of the long-term prediction; (b) It shows the pre-emphasis coefficient in the MFCC feature datasets is 0.9 of the long-term prediction.

The second neural network method used in this experiment is the long short-term memory (LSTM) algorithm. The experimental process presents different LSTM architectures, all based on two network hidden layers, respectively using 200, 300, 500 and 700 hidden neurons, using PCA for comparison. Table 4 lists the accuracy and training times of the four different number of hidden neural network layers with LPC and MFCC datasets, the LSTM training model network label layer = 2×200 indicates that there are 2 hidden layers containing 200 hidden neurons. Figs 21(A), 22(A), 23(A) and 24(A) show the classification process with LPC datasets while Figs 21(B), 22(B), 23(B) and 24(B) show the classification process after adding PCA method. Similarly, Figs 25–28 respectively show the similar illustrations as Figs 21–24 but with MFCC filtering algorithm. In addition, Figs 29 and 30 present, respectively, the two Feature datasets of the LPC and MFCC, where the long-term prediction of the LSTM algorithm has been added. The training set and test set occupy, respectively, 80% and 20% of the datasets. The reduced training time highlights the impact of PCA on LSTM calculations. The loss function with LPC datasets can show that PCA produces a smoother gradient descent process. In terms of time, PCA has a key impact on enhancing the advantages of LSTM algorithms. For the LSTM model, the accuracy of the LPC feature dataset increases with the number of hidden neurons. Introducing the PCA method increases the accuracy score and reduces the training period time. with increases from 200 to 700 hidden neuron structures resulting in sequential efficiency increases of 8.5%, 1.5%, 0.5%, and 0.2%. However, despite the significant decrease in the training period for the MFCC-PCA-LSTM, the accuracy of the MFCC feature datasets is slightly reduced, with increases from 200 to 700 hidden neurons producing sequential reductions in meta-architecture performance of -1.0%, -0.7%, -0.5%, and -0.2% in order. In other words, the MFCC-LSTM model can achieve a considerable degree of accuracy. In addition, as the number of hidden neurons increases, the LPC feature dataset gradually improves, while the MFCC feature dataset remains relatively unchanged. It can also be inferred from this that the number of hidden neurons will affect the accuracy score of the LPC model.

**Table 4:** Training results of LSTM models and PCA-LSTM models.

**Training model**	**LPC-LSTM**	**LPC-PCA-LSTM**	**MFCC-LSTM**	**MFCC-PCA-LSTM**
	**layer = 2**×**200**	**layer = 2**×**200**	**layer = 2**×**200**	**layer = 2×200**
Accuracy score	0.921	1.000	0.998	0.988
Accuracy difference ratio	8.5%		-1.0%	
Training period (sec.)	70.077	51.536	75.785	49.681
Training period difference ratio	-26.5%		-34.5%	
**Training model**	**LPC-LSTM**	**LPC-PCA-LSTM**	**MFCC-LSTM**	**MFCC-PCA-LSTM**
	**layer = 2**×**300**	**layer = 2**×**300**	**layer = 2**×**300**	**layer = 2×300**
Accuracy score	0.986	1.000	1.000	0.993
Accuracy difference ratio	1.5%		-0.7%	
Training period (sec.)	101.016	69.059	106.693	70.926
Training period difference ratio	-31.6%		-33.5%	
**Training model**	**LPC-LSTM**	**LPC-PCA-LSTM**	**MFCC-LSTM**	**MFCC-PCA-LSTM**
	**layer = 2×500**	**layer = 2×500**	**layer = 2×500**	**layer = 2×500**
Accuracy score	0.995	1.000	1.000	0.995
Accuracy difference ratio	0.5%		-0.5%	
Training period (sec.)	173.457	120.370	182.007	124.238
Training period difference ratio	-30.6%		-31.7%	
**Training model**	**LPC-LSTM**	**LPC-PCA-LSTM**	**MFCC-LSTM**	**MFCC-PCA-LSTM**
	**layer = 2**×**700**	**layer = 2**×**700**	**layer = 2**×**700**	**layer = 2×700**
Accuracy score	0.998	1.000	0.998	0.995
Accuracy difference ratio	0.2%		-0.2%	
Training period (sec.)	285.477	210.860	293.631	212.291
Training period difference ratio	-26.1%		-27.7%	

For the datasets constructed in this experiment, different neural network configurations will have different effects, and PCA increases the difference in performance, especially with LPC datasets. A significant performance improvement implies that, at the practical application level, this feature dataset faces many unexpected external factors.

**Fig 31:**
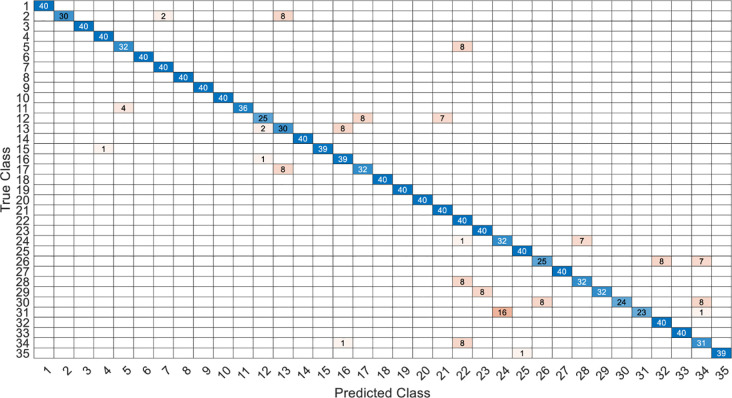
The confusion matrix of DNN-12-layer model with LPC datasets.

**Fig 32:**
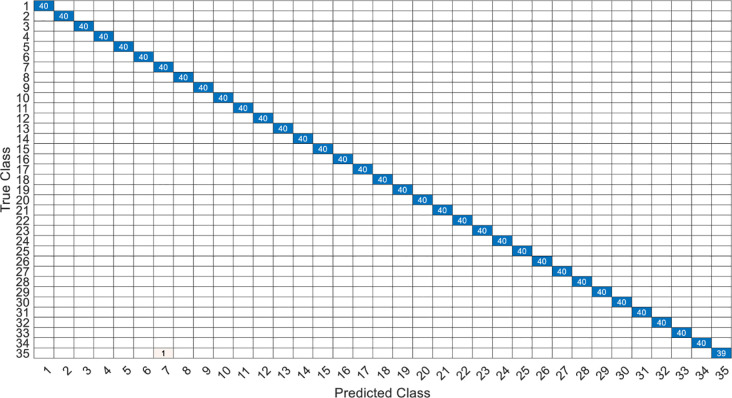
The confusion matrix of PCA-DNN-12-layer model with LPC datasets.

**Fig 33:**
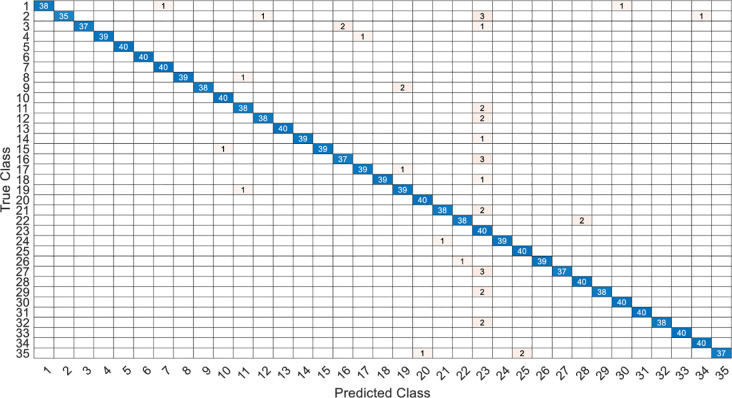
The confusion matrix of DNN-12-layer model with MFCC datasets.

**Fig 34:**
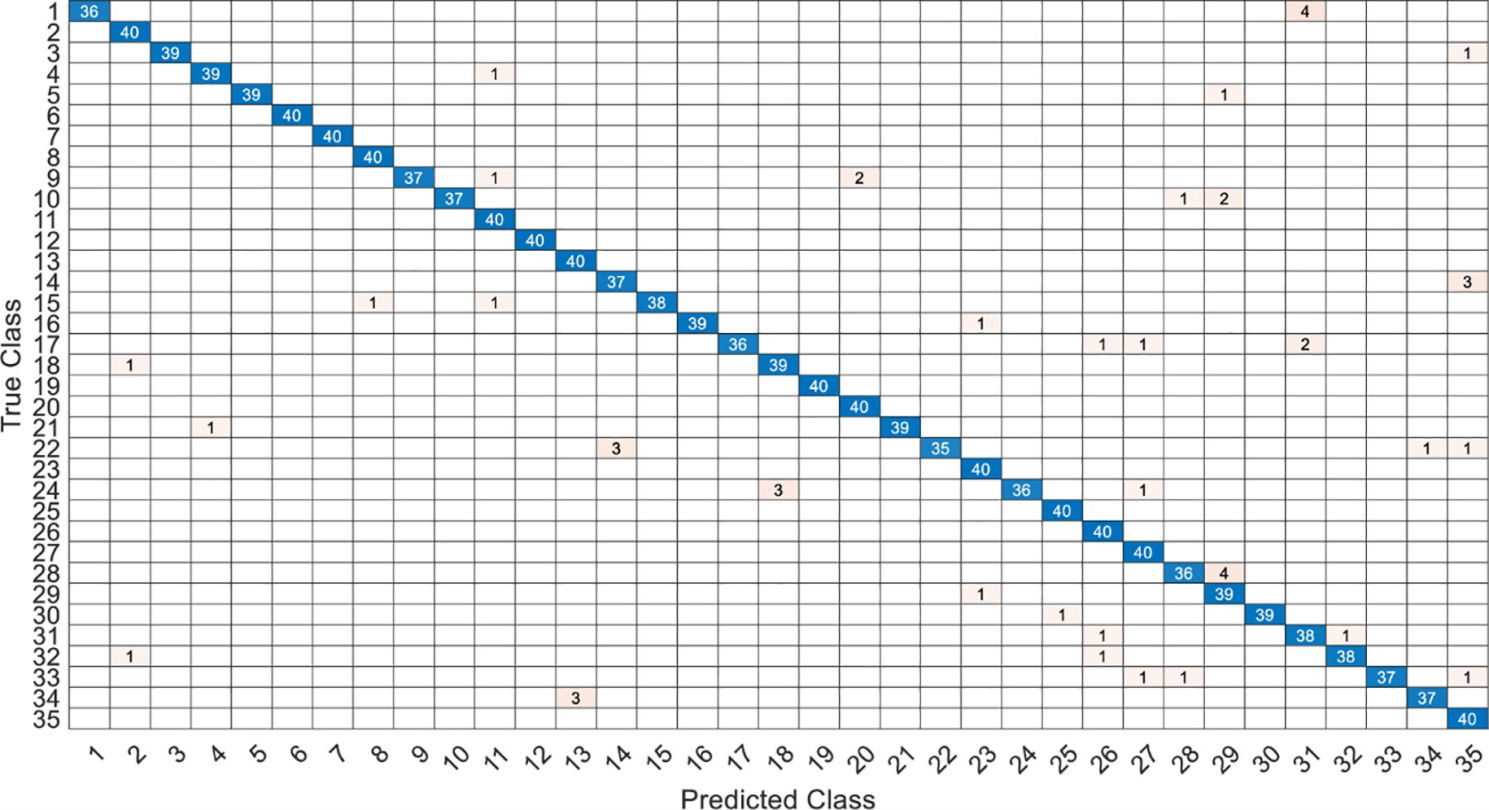
The confusion matrix of PCA-DNN-12-layer model with MFCC datasets.

**Fig 35:**
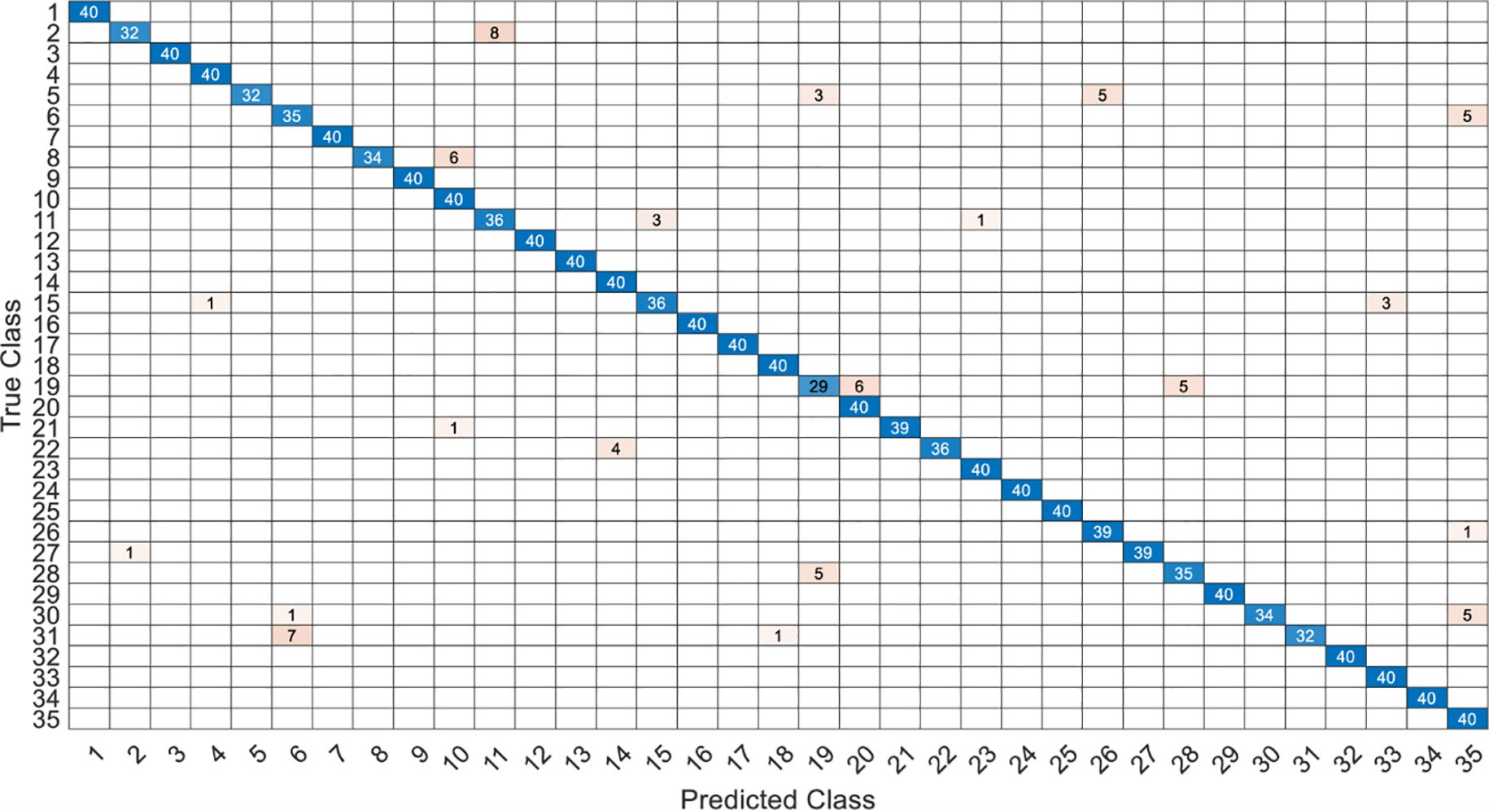
The confusion matrix of LSTM-2×200 model with LPC datasets.

**Fig 36:**
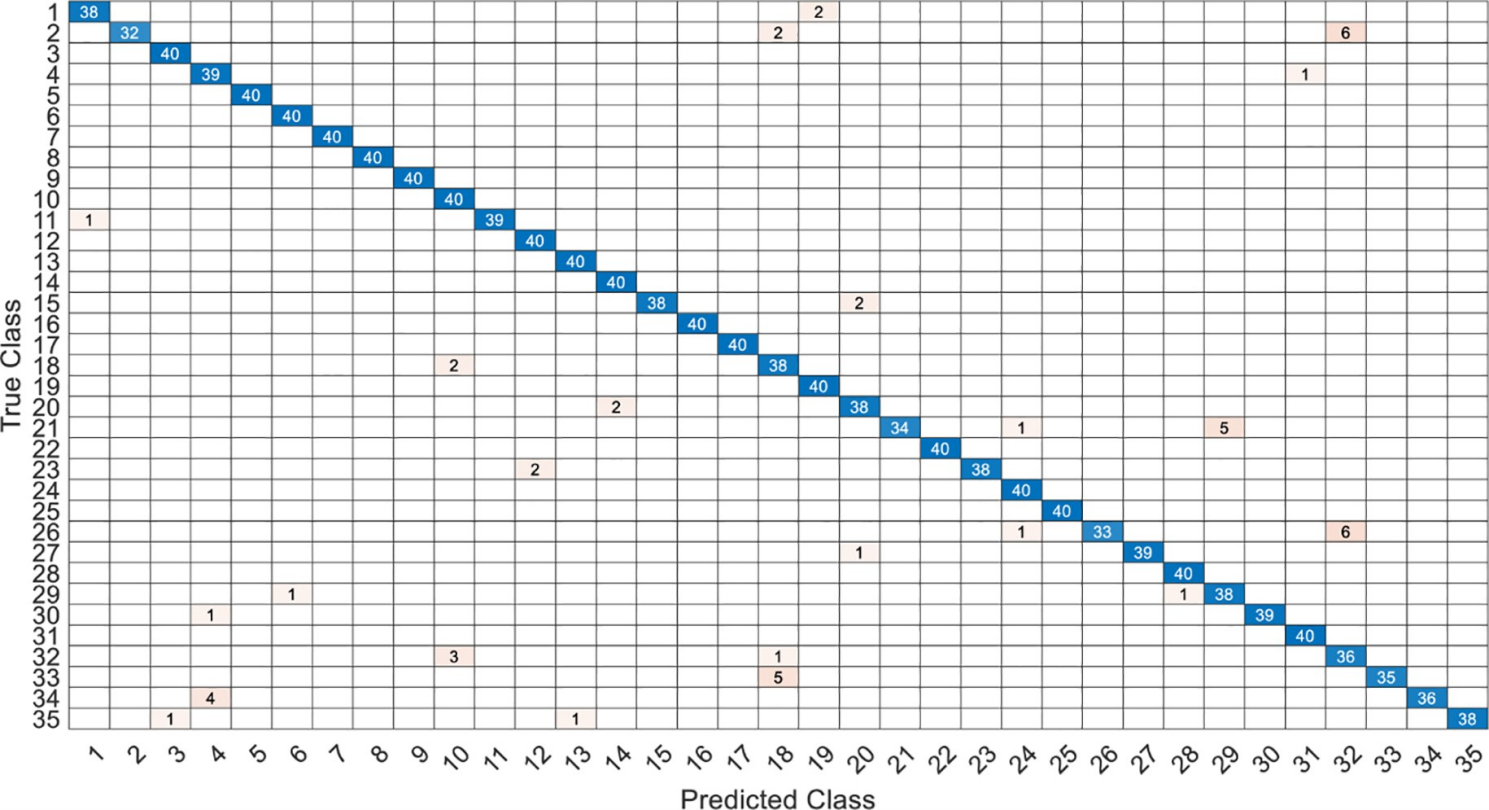
The confusion matrix of PCA-LSTM-2×200 model with LPC datasets.

**Fig 37:**
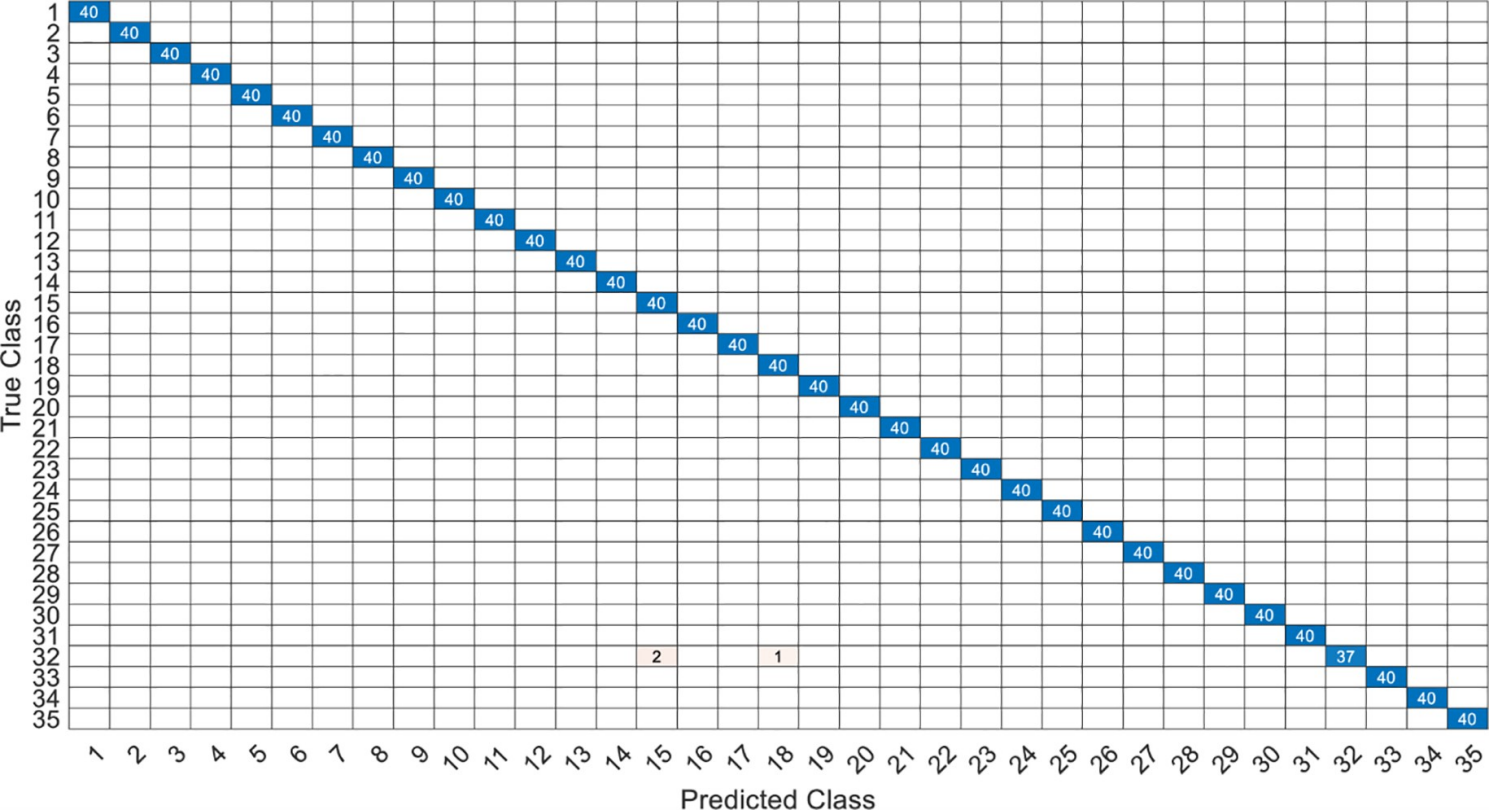
The confusion matrix of LSTM-2×200 model with MFCC datasets.

**Fig 38:**
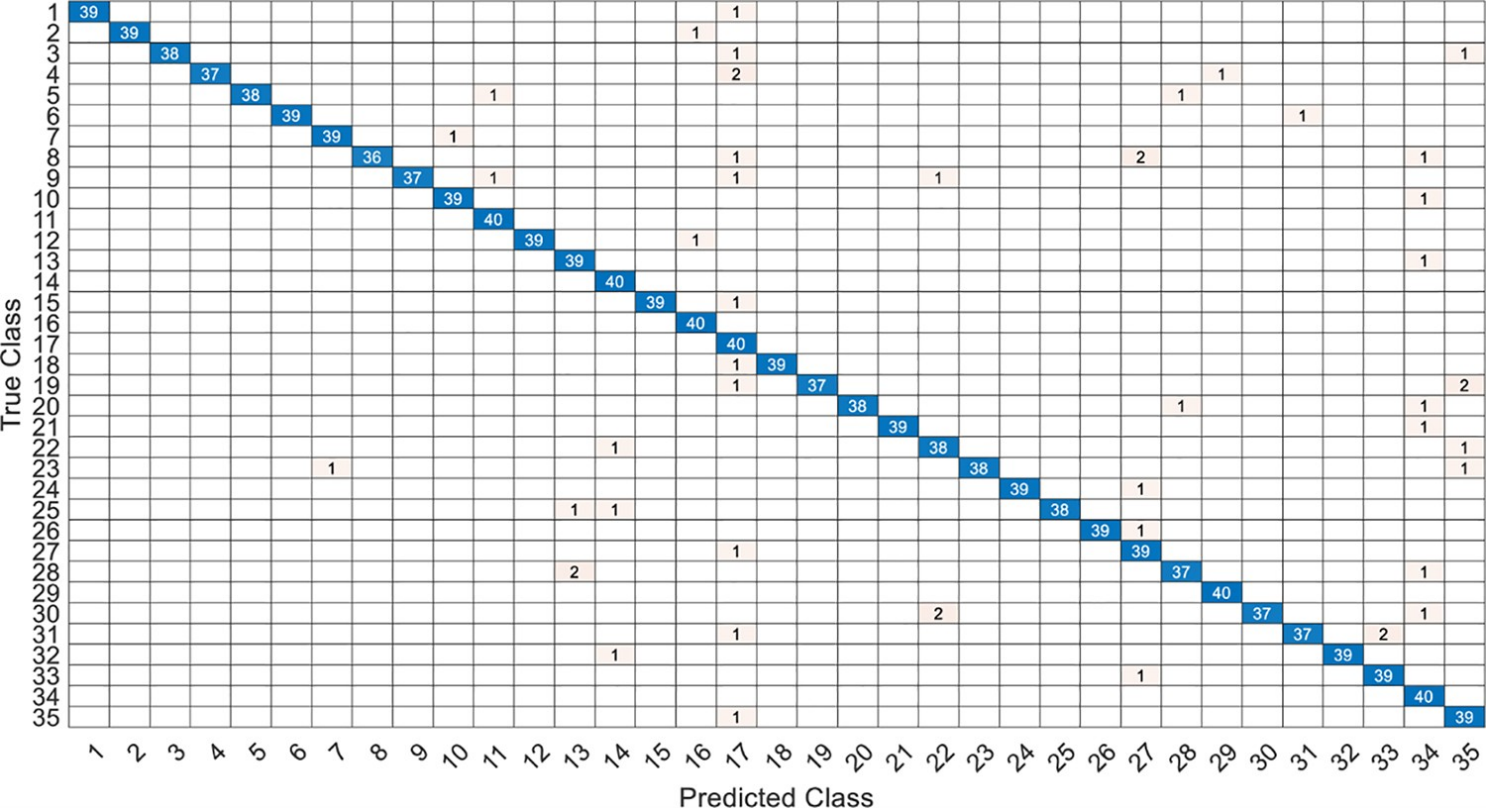
The confusion matrix of PCA-LSTM-2×200 model with MFCC datasets.

**Fig 39:**
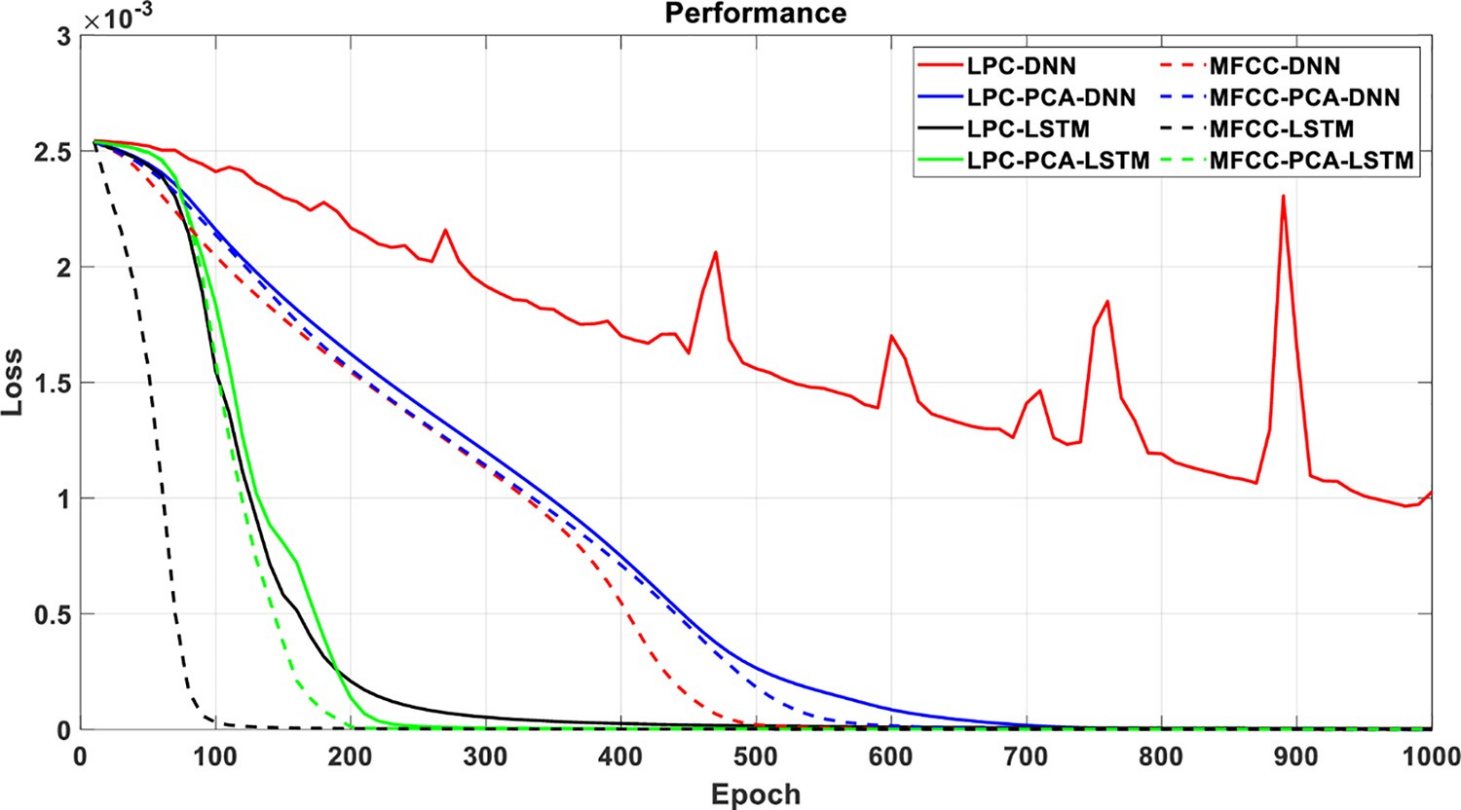
The loss function of network structures, LPC-DNN, LPC-PCA-DNN, LPC-LSTM, LPC-PCA-LSTM, MFCC-DNN, MFCC-PCA-DNN, MFCC-LSTM and MFCC-PCA-LSTM, as the epoch increases. 20-layer structure is selected in DNN while 1200-hidden neurons with 2 layers is set in LSTM. It seems that MFCC-LSTM model needs only 100 epochs to let the loss function converge completely, which can also save the training period.

This article specifically discusses the efficiency and calculation time through several models, and further analyzes the best algorithm combination. Table 5 shows the average score of the k-fold cross validation. Figs 31–34 present the feature datasets of the LPC and MFCC along with the obtained results of the confusion matrix from, respectively, the DNN and PCA-DNN. Figs 35–38 show the feature datasets of the LPC and MFCC, where the results of the confusion matrix are obtained by means of the LSTM and PCA-LSTM, respectively. Table 6 lists the four specific algorithm combinations. In terms of accuracy, all provide high-precision recognition effects. Different deep learning algorithms have different configuration architectures, along with different accuracy score presentations and training periods. In addition, Fig 39 shows that, compared with the DNN model, the LSTM model produces very fast gradient descent convergence within 300 epochs and the fastest gradient descent is found in the MFCC-LSTM model, which can converge within 200 epochs.

**Table 5:** Average score of 5-fold cross validation results of proposed models.

5-fold cross validation	LPC datasets	PCA-LPC datasets	MFCC datasets	PCA-MFCC datasets
DNN-12-layer	0.9007	0.9993	0.9700	0.9643
DNN-16-layer	0.8636	0.9964	0.9529	0.9500
DNN-20-layer	0.7350	0.9843	0.9464	0.9329
DNN-24-layer	0.4593	0.9900	0.9436	0.9029
LSTM-200*2 layer	0.9486	0.9629	0.9979	0.9643
LSTM-300*2 layer	0.9886	0.9764	0.9943	0.9750
LSTM-500*2 layer	0.9714	0.9793	0.9929	0.9729
LSTM-700*2 layer	0.9936	0.9864	0.9921	0.9821

**Table 6:** Loss functions between PCA-DNN model and PCA-LSTM model.

Network structure	LPC-PCA-DNN	LPC-PCA-LSTM	MFCC-PCA-DNN	MFCC-PCA-LSTM
Accuracy score	Great	Great	Great	Great
Training period (for 1000 epoch)	Short	Medium	Short	Medium
Gradient decay of loss function	Relatively slow	Relatively fast	Relatively slow	Relatively fast

This study is inspired from the feature classification experiments in [16]. The methods in [16] are to use the MFCC digital filtering algorithm to extract features from the original acoustic signals every single specy of the amphibian. The methods in [16] adjust the pre-emphasis coefficients to create multiple filtering effects, collect the feature spectral values, and construct the training datasets. Two widely used deep learning algorithms (DNN and LSTM) are applied to the classification model. The feature DSP in [16] is MFCC, where this study investigates LPC and MFCC. The platform is also different. In [16], Matlab is used, where Python Pytorch has been chosen for this study. With regards to the classification, MLP and SVM are used for the work in [16], as the title, where DNN and LSTM are used in this study. Moreover, this work possesses 20 more types of sound samples.

## 4. Conclusions

This research applies two algorithm architectures, DNN and LSTM, for feature classification of amphibian sounds through the bioacoustic spectrum. The machine learning structure used is the key to determining feature extraction and classification recognition performance. Available sound data is first collected for analysis by applying the LPC and MFCC algorithms for digital filtering of the data. The characteristic acoustic spectrum values obtained from filtering are then collected and respectively aggregated to construct synthetic datasets. The DNN as well as LSTM are the classifiers that use the number of hidden layers, different parameters, and function settings to analyze the effect and determine the optimal algorithm combination. The experimental results are presented in graphs and tables. Strikingly different classification results are obtained using the GPU with adaptive moment estimation algorithm (Adam) optimizer function. Results clearly show that the PCA algorithm can effectively reduce dataset dimensionality to achieve better classification and identification results for LPC datasets, indicating that this PCA algorithm provides improved recognition performance with LPC datasets. However, for MFCC datasets, there is no obvious benefit to importing the PCA method. This result shows that PCA has a greater impact on LPC datasets, but no impact on MFCC. In short, in the training of machine learning models, deep learning neural networks have been shown to be applicable for the processing and analysis of big data models and can achieve reasonable classification results through the use of effective classifier algorithms and training models with reasonable characteristics to identify specific species. Based on the research data and analytical results in this study, it is concluded that MFCC-LSTM not only possess high precision, but also have more benefit in reducing time during training models.

Future research can focus on applying other modern machine learning methods and algorithms. The widespread use of acoustic features would establish a key milestone in the improvement of modern technologies. The experiments presented here focus on the classification of animal acoustic features, but these techniques can be further used in the detection of abnormal sounds in human physiology, which would present a significant development in the use of sound analysis for medical diagnosis [50, 51].
